# Venlafaxine’s therapeutic reference range in the treatment of depression revised: a systematic review and meta-analysis

**DOI:** 10.1007/s00213-023-06484-7

**Published:** 2023-10-19

**Authors:** X.M. Lense, C. Hiemke, C.S.M. Funk, U. Havemann-Reinecke, G. Hefner, A. Menke, R. Mössner, T.G. Riemer, M. Scherf-Clavel, G. Schoretsanitis, G. Gründer, X.M. Hart

**Affiliations:** 1 Department of Molecular Neuroimaging, Medical Faculty Mannheim, Central Institute of Mental Health, University of Heidelberg, Heidelberg 68159 University J5, Mannheim, Germany; 2 Clinic of Psychiatry and Psychotherapy, University of Mainz, Mainz, Germany; 3 Arbeitsgemeinschaft für Neuropsychopharmakologie und Pharmakopsychiatrie (AGNP), Working Group “Therapeutic Drug Monitoring”, Munich, Germany; 4 Institute of Clinical Pharmacology and Toxicology, Charité University, Berlin, Germany; 5 Clinic of Psychiatry and Psychotherapy, University of Göttingen, Göttingen, Germany; 6 Vitos Clinic of Forensic Psychiatry, Eltville, Germany; 7 Psychosomatic Clinic Medical Park Chiemseeblick, Bernau a. Chiemsee, Germany; 8 Department of Psychiatry and Psychotherapy, University Hospital, Ludwig Maximilian University of Munich, Munich, Germany; 9 Department of Psychiatry and Psychotherapy, University Hospital of Tübingen, Tübingen, Germany; 10 Department of Psychiatry, Psychosomatics and Psychotherapy, University Hospital of Würzburg, Würzburg, Germany; 11 Department of Psychiatry, Psychotherapy and Psychosomatics, University Hospital of Zürich, Zürich, Switzerland

**Keywords:** Antidepressant, Depression, Venlafaxine

## Abstract

**Introduction:**

The selective serotonin and norepinephrine reuptake inhibitor venlafaxine is among the most prescribed antidepressant drugs worldwide and, according to guidelines, its dose titration should be guided by drug-level monitoring of its active moiety (AM) which consists of venlafaxine (VEN) plus active metabolite O-desmethylvenlafaxine (ODV). This indication of therapeutic drug monitoring (TDM), however, assumes a clear concentration/effect relationship for a drug, which for VEN has not been systematically explored yet.

**Objectives:**

We performed a systematic review and meta-analysis to investigate the relationship between blood levels, efficacy, and adverse reactions in order to suggest an optimal target concentration range for VEN oral formulations for the treatment of depression.

**Methods:**

Four databases (MEDLINE (PubMed), PsycINFO, Web of Science Core Collection, and Cochrane Library) were systematically searched in March 2022 for relevant articles according to a previously published protocol. Reviewers independently screened references and performed data extraction and critical appraisal.

**Results:**

High-quality randomized controlled trials investigating concentration/efficacy relationships and studies using a placebo lead-in phase were not found. Sixty-eight articles, consisting mostly of naturalistic TDM studies or small noncontrolled studies, met the eligibility criteria. Of them, five cohort studies reported a positive correlation between blood levels and antidepressant effects after VEN treatment. Our meta-analyses showed (i) higher AM and (ii) higher ODV concentrations in patients responding to VEN treatment when compared to non-responders (*n* = 360, *k* = 5). AM concentration-dependent occurrence of tremor was reported in one study. We found a linear relationship between daily dose and AM concentration within guideline recommended doses (75–225 mg/day). The population-based concentration ranges (25–75% interquartile) among 11 studies (*n* = 3200) using flexible dosing were (i) 225–450 ng/ml for the AM and (ii) 144–302 ng/ml for ODV. One PET study reported an occupancy of 80% serotonin transporters for ODV serum levels above 85 ng/ml. Based on our findings, we propose a therapeutic reference range for AM of 140–600 ng/ml.

**Conclusion:**

VEN TDM within a range of 140 to 600 ng/ml (AM) will increase the probability of response in nonresponders. A titration within the proposed reference range is recommended in case of non-response at lower drug concentrations as a consequence of VEN’s dual mechanism of action via combined serotonin and norepinephrine reuptake inhibition. Drug titration towards higher concentrations will, however, increase the risk for ADRs, in particular with supratherapeutic drug concentrations.

**Supplementary Information:**

The online version contains supplementary material available at 10.1007/s00213-023-06484-7.

## Introduction

The selective serotonin and norepinephrine reuptake inhibitor (SNRI) venlafaxine (VEN) has been used in the treatment of major depressive disorder (MDD), generalized anxiety disorder, and panic disorder (Ratiopharm 2017; Keller et al. 2007; Bundesärztekammer (BÄK), K.B.K., Arbeitsgemeinschaft der Wissenschaftli- and c.M.F. (AWMF) 2022) since 1995. Its good safety and efficacy profile has led to a position among the most commonly prescribed antidepressant drugs worldwide (Cipriani et al. 2018). Maintenance dosing is recommended within a range from 75 to 225 mg/day divided into three or two daily oral doses, if administered as immediate release (IR) formulation, or orally once daily, if administered as extended-release (ER) retard formulation (Government of Canada, D.P.D.o 2012). Higher dosing of up to 375 mg/day is approved and possible (Ratiopharm 2017), but not recommended by national and international guidelines (Bundesärztekammer (BÄK), K.B.K., Arbeitsgemeinschaft der Wissenschaftli- and c.M.F. (AWMF) 2022; Kennedy et al. 2016). In low doses, VEN predominantly expresses serotonin reuptake–inhibiting effects, whereas in higher doses (≥ 150 mg/day), it also acts as a noradrenaline reuptake inhibitor. As a result, higher VEN doses have been discussed being more effective than lower doses (Eap et al. 2021). Dose escalation is a common practice in clinical routine and is also recommended by national treatment guidelines in case of nonresponse to initial treatment (Bundesärztekammer (BÄK), K.B.K., Arbeitsgemeinschaft der Wissenschaftli- and c.M.F. (AWMF) 2022; Gauthier et al. 2017). In consequence, an early switch from SSRIs towards medium to high doses of VEN is a treatment rationale in case of insufficient response to SSRIs (Tadić et al. 2016; Engelmann et al. 2021). Therapeutic drug-monitoring (TDM) guidelines recommend drug-level guided dosing for patients being treated with VEN with the second highest level of recommendation (Hiemke et al. 2018). In contrast, a recently published dose/response meta-analysis (Rink et al. 2022) that included data from 15 VEN studies found no benefit among higher dosing regimens. Earlier studies reported similar findings (Furukawa et al. 2019). These findings are however hardly surprising when taking VEN’s pharmacokinetic profile into account. VEN is mainly metabolized to an active metabolite O-desmethylvenlafaxine (ODV) via cytochrome P450(CYP)2D6. CYP2C9 and CYP3A4 also play a role in the metabolism (Otton et al. 1996; Fogelman et al. 1999). At steady state, the ODV shows two- to three-fold higher levels compared to its parent drug. Dose adjustments are recommended in cases of hepatic or renal dysfunction (Government of Canada, D.P.D.o 2012). Gender and age also affect blood levels (BLs) from administered doses (Sigurdsson et al. 2015). Overall, high interindividual variability determines the expression of widely differing drug exposures in patients with the same drug dose (Whyte et al. 2006; Shams et al. 2006; Hansen et al. 2017). For VEN, concentration-dependent treatment effects are best evaluated using active moiety (AM) blood levels, comprising VEN plus ODV (Hiemke et al. 2018). Against expectations from guideline recommendations, to our knowledge, a concentration/effect relationship has not been systematically explored for VEN yet (Eap et al. 2021). Furthermore, a dose-dependent pattern has been implied for several adverse reactions (ADRs), such as hypertension, anorexia, nausea, agitation, dizziness, somnolence, tremor, and sweating (Government of Canada, D.P.D.o 2012).

The aim of this study was to estimate the therapeutic reference range for the AM of VEN for the treatment of depressive disorders and to discuss the use of TDM for VEN in clinical routine practice. The first objective assessed evidence of a relationship between blood levels and VEN efficacy/ADRs. The second objective assessed evidence on the serotonin transporter (SERT)/noradrenaline transporter (NET) occupancy from neuroimaging studies. Moderating factors on drug blood levels are furthermore identified in the course of this study.

## Materials and methods

### Inclusion criteria

Both randomized controlled trials (RCTs) and uncontrolled studies reporting VEN, ODV, or AM concentrations in humans (serum or plasma), referred to herein as BLs, were eligible for inclusion. Reviews and meta-analyses investigating a concentration/efficacy relationship for VEN were also included. Three types of studies were identified: (i) studies referring to BL in relation to clinical effects, (ii) studies reporting BL in relation to pharmacokinetics, and (iii) studies examining BL in relation to SERT or NET occupancy as measured with molecular imaging technology. Studies were included regardless of VEN drug formulation, dosing schemes, or design. All psychiatric indications were included, however, only patients with depression were considered a representative patient sample in terms of the study outcome. Inclusion and exclusion criteria are fully listed in the data supplement (table S1).

### Study selection process

We performed a systematic literature search according to the Preferred Reporting Items for Systematic Reviews and Meta-Analyses (PRISMA) statement (Page et al. 2021) including quality control of studies (Hart et al. 2021) and a final grading of available evidence (Hart et al. 2021; Hasan et al. 2019). The initial search was carried out on October 15, 2020, and updated on January 30, 2023. We systematically searched the literature using MEDLINE via the PubMed interface, the Web of Science Core Collection, PsycINFO, and Cochrane Library databases. Search strategies used keywords relevant to VEN dose, blood concentration, TDM, positron emission tomography (PET)/ single photon emission computed tomography (SPECT), and clinical response (data supplement S2). No preset database search filters were used. Two independent reviewers (XML and XMH) performed a screening of the literature. Relevant papers were checked for eligibility in full text. In cases where a final decision could not be made based on the abstract alone, the full article was reviewed. Any disagreements between the two reviewers were resolved in a subsequent discussion. Both reviewers independently extracted the following information from each study: lead author, year, title, country, study design, number and details of subjects, diagnosis, mean dose ± standard deviation (SD), mean blood concentration ± SD, concentration range, clinical efficacy or side effect measures, and main outcomes. Additional data was requested by the corresponding author, whenever concentration data were not complete. The study is registered under PROSPERO number CRD42020218248.

Four reviewers (XL, GH, XH, and KW) independently performed quality assessments of TDM components for all included studies according to a previously published rating instrument (see data supplement S3 for details) (Hart et al. 2021). Two reviewers (CF and XH) rated the quality of efficacy cohorts of randomized controlled trials (RCTs) using the Cochrane risk-of-bias tool for randomized trials (RoB 2) (fig. S4 a and b). The level of evidence for a concentration/effect relationship of VEN was determined following our study protocol (Hasan et al. 2019). Criteria for quality assessment relate to VEN and its active metabolite ODV, if applicable.

### Qualitative and quantitative synthesis

Outcomes of interest for qualitative synthesis were reports of an association between VEN and/or ODV BL and either the antidepressant effect or side effects. Eligible reports could be qualitative or quantitative but require a structured clinical assessment by a rating scale (for details see table S5, S6, and S7). Associations between BLs and clinical effects could be continuous as well as categorical. Reports on the moderating factors’ daily dosages [mg/day], CYP genetic polymorphisms, sex, age, and bodyweight on VEN and ODV BL were extracted (table S8, S9, and S10). Studies reporting SERT or NET occupancy in relation to participant’s BLs including 50% effective concentration values (EC_50_) for VEN and the active moiety were evaluated. For quantitative synthesis, mean, standard deviation, median, and interquartile range of relevant BLs were assessed. Only studies using extended-release (ER) formulations and investigating depressive disorders were included in the quantitative analysis. Mean and standard deviation of concentration-dose-ratio (C/D) were assessed. Data was either extracted from the manuscript or, if individual values were given, was calculated manually according to the Cochrane Handbook. If multiple assessments over the course of the study were available, the latest BL measures were used for the analysis.

### Statistical analysis

A combined meta-analysis was performed using R version 4.0.3 “metafor and meta package” and Review Manager version 5.4. *I*^2^ statistic was used to evaluate the heterogeneity of the studies, with *I*^2^ values > 50% indicating heterogeneity. The 95% confidence intervals (CIs) were calculated from mean concentrations and C/D values, and data was combined using random-effect models based on the *I*^2^ statistic. Quality assessment criteria that could have a potential influence on the clinical validity of a reference range were identified a priori. The impact of these quality criteria as moderating factors on mean drug concentrations was investigated by subgroup analyses of studies rated sufficient or insufficient on those criteria. The following criteria were identified for quantitative subgroup analysis: Q1 “diagnosis depressive disorder”, Q2a “psychiatric comedication”, Q2b “CYP-interfering comedication”, Q3 “dose design”, and Q4 “age”. Subgroup comparisons were conducted if a minimum of three records per subgroup were available. Forest plots of subgroup differences identified as significant (*p* ≤ .05) were retrieved for visualization of subgroup differences. Linear regression analysis was used to display the relationship between VEN dose and active moiety BLs. To quantitatively investigate the relationship between antidepressant concentration and efficacy, an overall meta-analysis of differences in the antidepressant concentration between responders and nonresponders was conducted via RevMan (Version 5.4.1) (Collaboration TC 2020) using standardized mean differences and Hedge’s g, as effect estimate in a random effects model to account for assumed between-study heterogeneity. A leave-one-out meta-analysis to investigate the influence of each study on the overall effect-size estimate was performed.

## Results

### Study overview

In total, 1168 studies were initially identified through database searching; five records were detected through other sources (see data supplement S3 for PRISMA flow chart). Seventy articles assessing 62 study cohorts met the inclusion criteria including 29 concentration/effect studies, 29 concentration studies, and four neuroimaging studies. No meta-analysis investigating VEN’s concentration/effect relationship was found. Diagnoses varied among studies and included patients with MDD/depressive symptoms, obsessive compulsive disorder (OCD), attention deficit hyperactivity disorder (ADHD), and psychotic disorders (table S2-S4). General quality criteria for the TDM component were assessed for all 68 studies. Most studies investigated a representative patient sample (*k* = 48, *n*(VEN) = 14, 574; Q1) apart from the neuroimaging studies that were included by reason of the inclusion of healthy subjects. A relevant diagnosis classification system and heterogeneous sample in terms of diagnosis were used in most concentration/effect studies (*k* = 23, 77%), whereas most concentration studies due to their naturalistic design did not fulfill this criterion (*k* = 3, 8.8%). Most studies used a flexible dose design (*k* = 58; Q2) and allowed for or provided insufficient information on relevant psychiatric or CYP2C19-interfering comedication (*k* = 52; Q3). Sampling at steady state (Q6a) was performed in 57 studies, and most of them described the use of an adequate analytical method (Q5) and investigated a sufficiently broad concentration range (Q7b) (both met by 52 studies). An adequate sampling time (Q6b) was met by 47 studies. More than 75% of those studies reported a sampling time right before the next drug administration at the trough level. Overall, a high quality of TDM was found in most studies. Cross-sectional studies (CSS) usually did not consider repeated sampling time points, whereas most of the other studies did (Q7a).

### Relationship between drug levels and antidepressant efficacy after oral venlafaxine administration

**Fig. 1:**
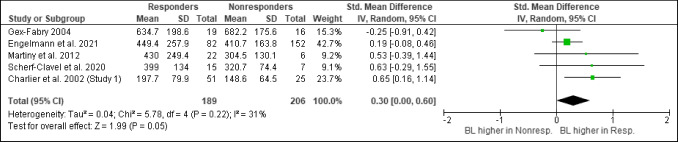
ROC-analysis o-desmethylvenlafaxine blood levels [ng/ml] using data from Engelmann et al. (2021) (AUC-value [95% CI] = 0.595 [0.517, 0.672]; *p* = 0.017)

A total of 27 studies collected data on VEN efficacy in patients with a depressive disorder. Four studies reported a positive (Hoencamp et al. 2000; Scherf-Clavel et al. 2020; De Donatis et al. 2021; Charlier et al. 2002; Stamm et al. 2014) and three studies a negative relationship between VEN efficacy and BL (Schoretsanitis et al. 2019; Berm et al. 2016) (Table 1). 14 studies did not find any relationship. One of the four studies reporting a positive relationship was a cohort study using a fixed dosing regimen (Stamm et al. 2014). The study, considered at low risk of bias (TDM score 7/10; study score 7/10), showed significantly higher ODV BLs in responders (≥ 50% HAM-D/HDRS reduction) compared to non-responders (Stamm et al. 2014). No differences between responders and non-responders were reported for VEN or active moiety BLs. A combination of early improvement (≥ 20% HAM-D reduction) and high ODV level was found to be a predictor for treatment response in this sample. A limitation of the study was the high drop-out rate of over 50%. The three other studies that found a positive relationship were cohort studies using a flexible dosing scheme; all of them reported a positive correlation between the active moiety and antidepressant effects (Scherf-Clavel et al. 2020; De Donatis et al. 2021; Charlier et al. 2002). Charlier and colleagues defined antidepressant response as a decrease of at least 50% in MADRS total score from baseline (moderate risk for bias; TDM score 8/10; study score 6/10) (Charlier et al. 2002). Scherf-Clavel and colleagues found a linear relationship between active moiety BL and HAMD-21 reduction in a naturalistic sample of 36 MDD patients (moderate risk for bias; TDM score 6/10; study score 5/10) (Scherf-Clavel et al. 2020). Patients with active moiety BL above 400 ng/ml showed larger clinical improvement from baseline than patients below this threshold (not significant after correction for multiple testing). Using ROC analysis, a threshold for remission was reported (393 ng/m). De Donatis and colleagues found a curvilinear relationship between active moiety BLs and response after 1 and 3 months of continuous treatment after excluding patients with non-detectable VEN BLs and patients with BLs above 800ng/ml (low risk for bias; TDM 8/10; study score 9/10) (De Donatis et al. 2021). The authors suggested a maximum of the antidepressant effects at about 400 ng/ml active moiety BLs. A reanalysis of data from a study by Engelmann and colleagues (TDM 7/10; RoB low) revealed an additional positive relationship that has not been described before. Higher ODV, but not VEN or active moiety BLs were found in responders (*p* = 0.017, Fig. 1) (Engelmann et al. 2021). The patient sample comprised patients with depression who were switched to VEN after insufficient response to a previous escitalopram treatment. The ODV interquartile range (IQR; 25–75%) across responders (50% reduction in HAM-D score) was 215–380 ng/ml. ROC analysis revealed a threshold of 289 ng/ml indicating a response (Fig. 1) (Engelmann et al. 2021).

**Table 1:** Findings on the concentration/effect relationship for venlafaxine, o-desmethylvenlafaxine, and the active moiety in single studies

Author, year	Design	Efficacy measure	Dose design	BLs below range	Concentration/ effect relationship	Implication for therapeutic reference range
De Donatis et al. (2021)	CS, *N* = 52	HAMD-21 % improvement	Flexible	PY	Positive continuous (u-shaped)	Confirms current AM range of 100–400 ng/ml
Scherf-Clavel et al. (2020)	CS, *N* = 23	HAMD-21 % improvement	Flexible	N	Positive continuous (linear)	ROC predicts remission (HAMD ≤ 7) above 393 ng/ml for AM
Charlier et al. 2002	CS, *N* = 22	MADRS total score	Flexible	Y	Positive continuous (linear)	Suggested range: 125–400 ng/ml for AM
Engelmann et al. (2021)	RCT, *N* = 119	HAMD score reduction	Flexible	Y	Positive dichotomized	ROC predicts remission (HAMD < 50%) above 289 ng/ml for ODV, not for VEN
Hoencamp et al. (2000)	CS, *N* = 37	HAMD-17, MADRS	Fixed	PY	Negative continuous (linear)	VEN only, not for ODV at week 7
Schoretsanitis et al. (2019)	CSS, *N* = 858	CGI-S	Flexible	Y	Negative dichotomized	-
Berm et al. (2016)	RCT, *N* = 40	MADRS, HAMD score reduction	Flexible	Y	Negative dichotomized	-
Stamm et al. (2014)	CS, *N* = 204	HAMD score reduction	Fixed	Y	Positive dichotomized	ODV only, not for VEN or AM

*CSS* cross-sectional study design, *CS* cohort study design, *RCT* randomized controlled study design, *Y* yes, *N* no, *PY* probably yes

A negative correlation between antidepressant effects and BLs of VEN and the active moiety, but not ODV levels was reported in patients with MDD by Hoencamp and colleagues (Hoencamp et al. 2000). This cohort study used a fixed dosing design (TDM score 8/10; study score 4/10), and the response was assessed as a decrease in HAM-D and MADRS total score from baseline (Hoencamp et al. 2000; Veefkind et al. 2000). Two flexible dosing studies reported a negative correlation between concentration and clinical effects after dichotomizing the patient sample into those with AM concentrations ≤ 400 ng/ml and > 400 ng/ml (Schoretsanitis et al. 2019; Berm et al. 2016). One of them followed a cross-sectional, naturalistic design and was rated with a high risk for bias (TDM 5/10; study score 2/8) also due to an exclusion of a certain patient group (patients with daily doses below 100 mg/day) from the analysis. More than 85% of the subjects in this study were non-responders to VEN. The second flexible-dose study constituted a post-hoc analysis of a sample from an RCT (TDM 5/10; RoB low) comprising patients 60 years and older receiving concomitant psychotropic comedications; active moiety BLs above 400 ng/ml were related to higher rates for non-response (Kok et al. 2007).

#### Quantitative synthesis

**Fig. 2:**
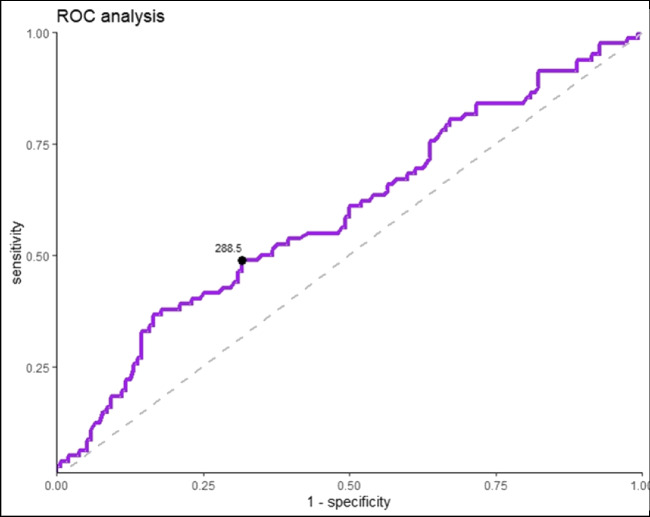
Mean venlafaxine active moiety blood levels [ng/ml] in responders and non-responders (*k* = 5)

Quantitative synthesis was performed with five eligible studies (*n* = 395). From seven studies providing sufficient data, one study each was excluded due to (i) an implausible ratio (Schoretsanitis et al. 2019) and (ii) the incomplete inclusion of responders and nonresponders (Veefkind et al. 2000). The combined effect estimate when comparing responders’ and non-responders’ drug concentrations in adult patients with a depressive disorder was significant (EE = 0.30 [0.00, 0.60], p ≤0.05, Fig. 2). Hedges’ g indicates higher active moiety BLs in responders (*n* = 189; mean ± SD = 412 ± 208 ng/ml) when compared to non-responders (*n* = 206; mean ± SD = 372 ± 139 ng/ml).

To sum up, three well-conducted cohort studies reported a positive relationship between antidepressant effects and active moiety BLs. One cohort study at low risk of bias reported a positive correlation between antidepressant effects and ODV BLs. Some studies of moderate to high risk for bias reported contradictory findings. The meta-analysis was able to show a positive concentration/efficacy relationship for the active moiety. Overall, the findings support a limited level of evidence (level B; limited) for the concentration/effect relationship of VEN’s active moiety.

### Relationship between drug levels and adverse drug reactions (ADRs) after oral venlafaxine administration

Eleven studies assessed ADRs in patients with depressive disorders during VEN treatment. Six studies reported a relationship between BLs (*n* = 2) or different genotypes (*n* = 4) and the occurrence of specific ADRs, whereas five studies did not find a clear relationship. One study comprised a patient cohort nested in an RCT (TDM 7/10; RoB low). The authors reported that patients with active moiety BLs above the therapeutic reference range of 400 ng/ml suffered more often from ADRs. However, differences reached significance for the ADR tremor only (Engelmann et al. 2021). Another RCT investigating children and adolescents found active moiety exposure over the geometric mean of 231.8 ng/mL being associated with orthostatic dizziness, cardiovascular, and dermatologic adverse effects (Sakolsky et al. 2011). Two cohort studies found that VEN-induced akathisia (ABCB1 polymorphism) (Ozbey et al. 2017) and side effects affecting skin, sexual function, and breast tissue occurred more often in specific genotypes (CYP2D6) (Whyte et al. 2006). A third cohort study found a positive correlation between the number of ADRs, measured with the UKU scale, as well as lower sodium levels and lower MPRs (Shams et al. 2006). Last, one last RCT (Lobello et al. 2010) reported an increase in the occurrence of increased alkaline phosphatase, sweating, and insomnia in CYP2D6 poor metabolizers (PM) compared to extensive metabolizers (EMs). Metabolizer phenotypes were determined based on the ratio of ODV to VEN concentrations (metabolic ratio). In conclusion, one study supported a low grade of evidence for concentration-dependent tremor after VEN treatment at higher concentrations in adults (level C; low). Multiple genotyping studies suggested further plausible concentration-dependent side effects due to an altered metabolism of VEN in specific patient groups. However, a clear relationship between the risk of VEN-related ADRs and BLs cannot be derived from these studies.

### Findings from neuroimaging studies

Four neuroimaging studies investigated SERT (*k* = 3, *n* = 42) or NET (*k* = 1, *n* = 21) occupancy in subjects treated with VEN (Frankle et al. 2018; Arakawa et al. 2019a; Meyer et al. 2004; Shang et al. 2007) (data supplement, table S8). One PET study by Frankle and colleagues scanned healthy male volunteers treated with escalating dose regimens. The occupancy curve suggested that no less than 85 ng/ml ODV resulted in an occupancy of 80% of SERT (EC_80_) (Frankle et al. 2018). An occupancy curve for VEN was reported by Meyer and colleagues (Meyer et al. 2004) with 14 ng/ml relating to 80% SERT occupancy. Since ODV concentration was on average two- to three-fold higher, an EC_80_ of 28–42 ng/ml can be assumed. The predicted EC_80_ for the active moiety would be 42–56 ng/ml. One PET study investigated NET occupancy after VEN treatment. The findings suggested that 50% NET occupancy would be reached at high active moiety BLs of at least 670 ng/ml (Arakawa et al. 2019a).

### Population-based target ranges and moderating factors on venlafaxine BLs

**Fig. 3:**
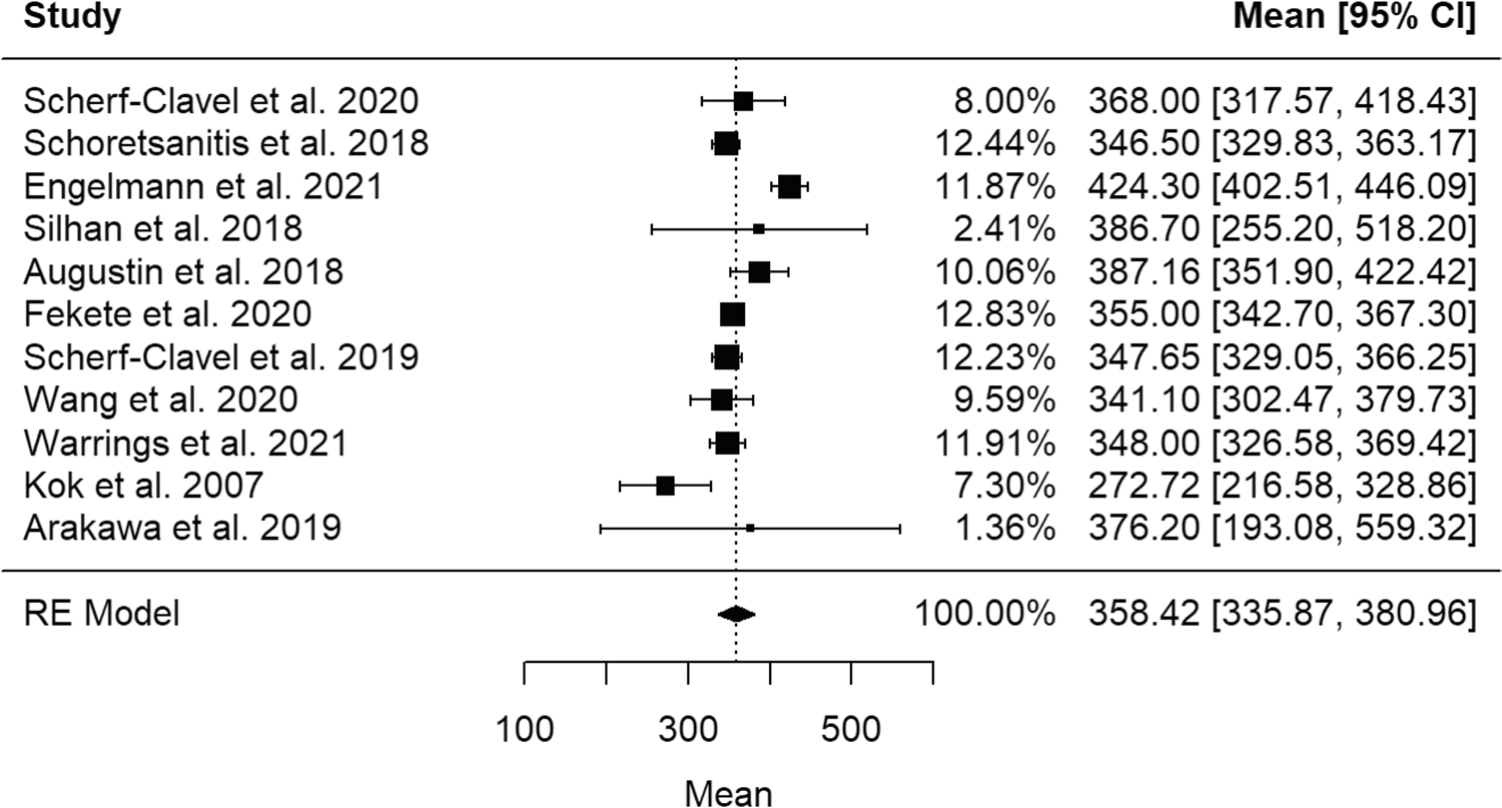
Forest plot–combined AM [ng/ml] from 11 studies with given median

Data to compute preliminary target ranges (25th–75th interquartile range) was available from 11 studies (*n* = 3200) (Engelmann et al. 2021; Scherf-Clavel et al. 2020; Schoretsanitis et al. 2019; Kok et al. 2007; Silhan et al. 2019; Augustin et al. 2018; Fekete et al. 2020; Scherf-Clavel et al. 2019; Wang et al. 2020; Warrings et al. 2021; Arakawa et al. 2019b) (Fig. 3). For the active moiety a range between 225 and 450 ng/ml was calculated (mean 358 ± 202 [336, 381] ng/ml, *Q* = 52.2; df = 10; *I*^2^ = 85.8%; *T*^2^ = 991.5). For VEN, studies revealed an interquartile range of 45 to 163 ng/ml (mean 128 ± 136 [111, 145] ng/ml, *Q* = 153.1; df = 10; *I*^2^ = 89.9%; *T*^2^ = 587.9). An interquartile range for ODV of 144–302 ng/ml was computed (mean 223 ± 133 [204, 242] ng/ml, *Q* = 86.5; df = 10; *I*^2^ = 92.9%; *T*^2^ = 819.9).

#### Concentration/dose relationship

**Fig. 4:**
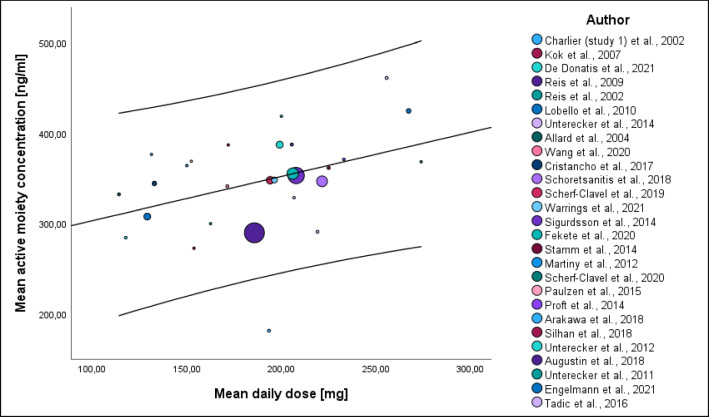
Linear regression analysis including all available studies (*N* = 8211, *y* = 254 + 0.49 * *x*, *r*2 =0.152, *p* = 0.04, *F* = 4.675, β-coefficient = 0.390 (0.024–0.952))

**Fig. 5:**
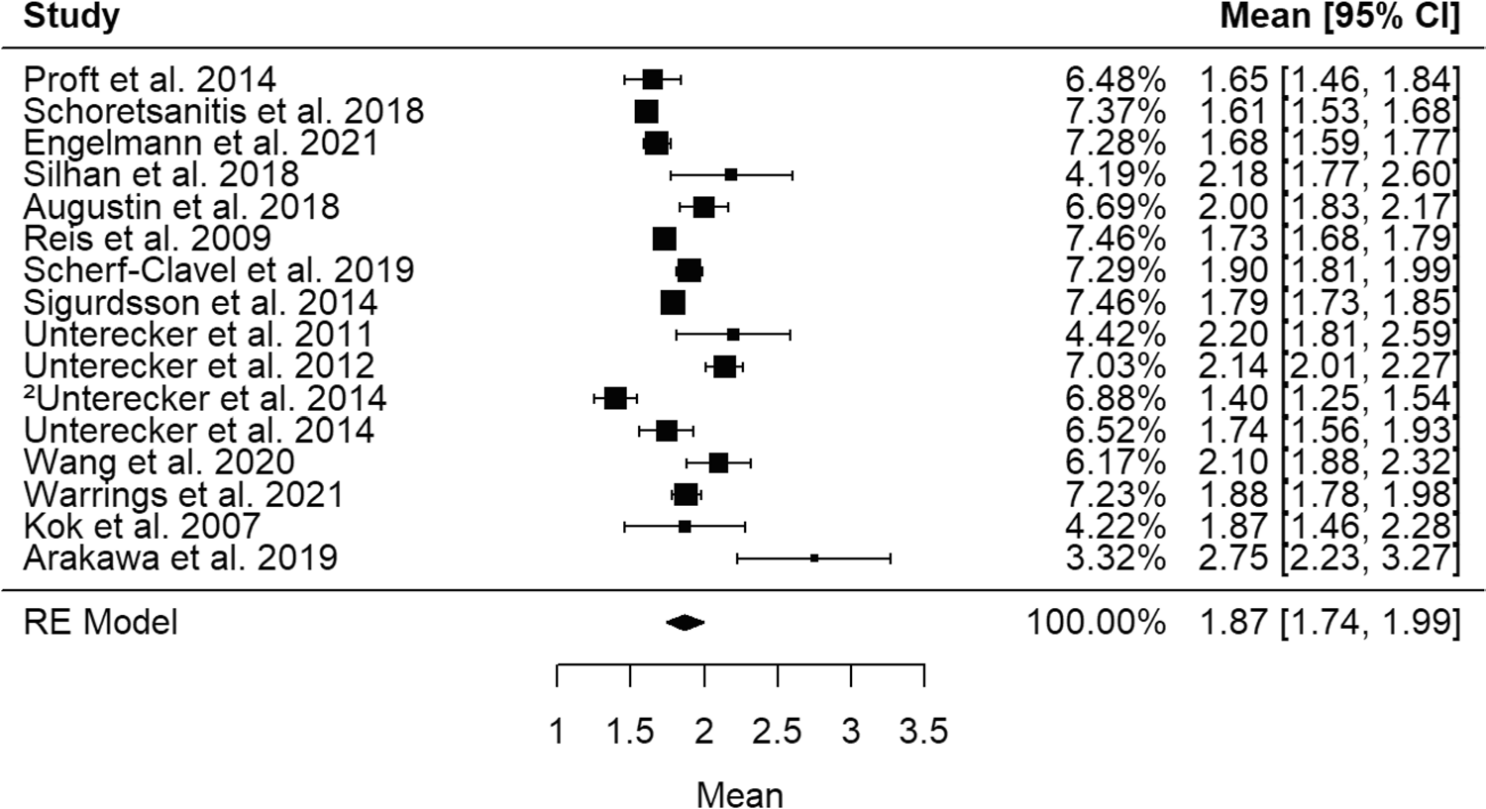
Forest plot–combined concentration-dose ratios [ng/ml/mg]

We identified 19 studies that performed correlation analyses on daily dose and VEN, ODV, or active moiety BLs. Most studies found a correlation between VEN oral doses and VEN BLs (*k* = 12) or ODV BL (*k* = 12) (Table 2). Nine studies reported a correlation for active moiety BLs. The linear regression analysis across 28 studies (*n* = 8211) that used VEN ER formulations found a strong relationship between daily dose and active moiety BLs (*r* = 0.564; *p* = 0.003) (Fig. 4). A mean daily dose of 188 mg/day resulted in a combined mean [CI 95%] active moiety BL of 344 ng/ml [322, 366]. For further analyses, only studies including patients with depressive disorders were considered. The combined mean active moiety C/D ratio across 16 studies (*N* = 6,117) is 1.87 [1.74, 1.99] ng/ml/mg/day (*Q* = 120.2; df = 14; *I*^2^ = 94.4%; *T*^2^ = 0.1, 95% confidence interval [CI]) (Fig. 5). The combined mean ODV C/D ratio across 11 studies (*n* = 2,751) is 1.13 [1.05, 1.21]) ng/ml/mg/day (*Q* = 104.3; df = 10; *I*^2^ = 89.3%; *T*^2^ = 0.01, 95% confidence interval [CI]). The combined mean VEN C/D ratio across 11 studies (*n* = 2,751) is 0.69 [0.60, 0.79] ng/ml/mg/day (*Q* = 88.1; df = 10; *I*^2^= 90.2%; *T*^2^ = 0.02). Subgroup analyses identified “age” as a potential moderator on the active moiety C/D ratio (*Q* = 133.7; df = 13; *p* < 0.0001), on active moiety BLs (*Q* = 51.5; df = 9; *I*^2^ = 0%; *T*^2^ = 0; *p* < 0.0001), and on ODV BLs (*Q* = 62.4; df = 9; *p* < 0.0001). Estimated drug levels from suggested maintenance doses are reported in Table 3.

**Table 2:** Findings on correlations between (i) dose, body weight/BMI, sex, age, co-medication and (ii) venlafaxine, o-desmethylvenlafaxine blood level

Author, year	Dose	Body weight	Sex	Age	Comedication
Denys et al. (2003) (*N* = 75)	(X)^1^				
Boulton et al. (2010) (*N* = 80)					**-** (Aripiprazole)
Martiny et al. (2012) (*N* = 31)					**-** (Pindolol)
Sakolsky et al. (2011) (*N* = 119)	**X**				
Aldosary et al. (2022) (*N* = 10)	**X**				
Gex-Fabry et al. (2002; 2004) (*N* = 35)			(**X**)^2^		
Charlier et al. (2002) (*N* = 119)	**X**				
Charlier et al. (2002) (*N* = 76)	**X**				
De Donatis et al. (2021) (*N* = 5)	**-**				
Grasmäder et al. (2004) (*N* = 17)					**X** (Lorazepam)
Grözinger et al. (2003) (*N* = 8)					**X** (Melperon)
Hoencamp et al. (2000) *N* = 60)	(X)^3^				**-** (Lithium)
Shams et al. (2006) (*N* = 100)	**X**				
Stamm et al. (2014) (*N* = 04)					**-**
Steen et al.(2015) (*N* = 5(2)	**X**				
Schoretsanitis et al. (2018) (D*2*) (*N* = 737)		**X**	**X**		
Sigurdsson et al. (2015) (*N* = 1417)	**X**	**X**	**X**	**X**	
Warrings et al. (2021) (*N* = 380)		**X**			
Waade et al. (2014) (*N* = (255)				**X**	
Fekete et al. (2020) (*N* = 953)			**X**	**X**	
Hansen et al. (2017) (*N* = 1077)	**X**		**X**	**X**	
Scherf-Clavel et al. (2019) (*N* = 534)			**X**	**X**	
Unterecker et al. (2012) (*N* = 478)	**X**		**X**	**X**	(**X**)^4^
Unterecker et al. (2014) (*N* = 8(2)					**X** (Valproate)
Wang et al. (2020) (*N* = 737)	**X**		**X**	**X**	**X** (Valproate), **X** (Clozapine)
McAlpine et al. (2011) (*N* = 95)	**X**		**X**		
Komahashi-Sasaki et al. (2020) (*N* = 75)	**X**		**X**		
Kringen et al. (2020) (*N* = 1000)				**X**	
Reis et al. 2002(*N* = 1781)	**X**			**X**	
Reis et al. (2002) (*N* = 187)	**X**			**X**	**X** (Alimemazine, Omeprazole)
Augustin et al. (2018) (*N* = 130)					**X** (Amlodipine, Ramipril)
Frankle et al. (2018) (*N* = 16)	**X**				
Kowalewski et al. (2019) (D(2) (*N* = 939)					**X** (Trimipramine)
Kuzin et al. (2018) (D2) (*N* = 986)					**X** (Pantoprazole, omeprazole)
Paulzen et al. (2015) (*N* = 16)	**X**				
Paulzen et al. (2018) (D2) (*N* = 1067)					**-** (Mirtazapine) **X** (Doxepine)

The letter (X) denotes relationship found; the hyphen (-) symbol denotes no relationship found

^1^Non-linear relationship

^2^Relationship regarding the (+)/(−) enantiomers was found

^3^Not defined if positive or negative relationship was found

^4^Not specified

**Table 3:** Expected venlafaxine concentration from mean C/D ratios after the administration of 75 mg, 150 mg, 225 mg, and 375mg venlafaxine ER

Venlafaxine dose per day	Expected active moiety BL (*N* = 6, 117) from CD 1.87 [1.74, 1.99]	Expected venlafaxine BL (*N* = 2, 751) from CD 0.69 [0.60, 0.79]	Expected o-desmethylvenlafaxine BL (*N* = 2, 751) from CD 1.13 [1.05, 1.21]
75 mg	140 [131, 149] ng/ml	52 [45, 59] ng/ml	85 [79, 91] ng/ml
150 mg	281 [261, 299] ng/ml	104 [90, 119] ng/ml	170 [158, 182] ng/ml
225 mg	421 [392,448] ng/ml	155 [135, 178] ng/ml	254 [236, 497] ng/ml
375 mg	701 [653, 746] ng/ml	259 [225, 296] ng/ml	424 [394, 454] ng/ml

#### Influence of age, sex, and body weight

Nine studies found significantly higher dose-corrected concentrations (C/D) of the active moiety, VEN, and/or ODV in elderly patients (Table 2). In the studies, elderly patient groups were defined with a minimum age of 59 to 65 years. Four studies described differences in dose-corrected active moiety BLs ranging from 50 to 61% (Sigurdsson et al. 2015; Hansen et al. 2017; Fekete et al. 2020; Waade et al. 2014) while two studies found up to 200% increases in VEN BLs (Hansen et al. 2017; Reis et al. 2002). Four studies showed differences in ODV ranging from 50 to 63% (Hansen et al. 2017; Fekete et al. 2020; Wang et al. 2020; Unterecker et al. 2012). Kidney function has been found frequently reduced in elderly patients. This is important as studies correlating kidney function and VEN BLs are limited. In the sense of evidence-based medicine, more clinical studies that focus on this issue are needed. Seven studies showed significantly higher C/D ratios of the active moiety in women when compared to men (Sigurdsson et al. 2015; Hansen et al. 2017; Fekete et al. 2020; Scherf-Clavel et al. 2019; Wang et al. 2020; Unterecker et al. 2012; Schoretsanitis et al. 2018). One of those studies showed an increase of 50% in 34 women compared to 57 men (Wang et al. 2020). Another study showed a difference of approximately 70% in C/D of the active moiety between females older than 65 (*n* = 176) and males younger than 65 (*n* = 465). Two studies showed significantly differing C/D ratios of ODV in women compared to men (Schoretsanitis et al. 2018; Komahashi-Sasaki et al. 2020). One of them showed an increase of CD/ODV by 219% in 450 women compared to 287 men (Schoretsanitis et al. 2018), while the other study showed a significant decrease of CD/ODV in 47 women when compared to 28 men (Komahashi-Sasaki et al. 2020). Three studies reported a negative correlation between BMI and C/D ratio of the active moiety (Sigurdsson et al. 2015; Warrings et al. 2021; Schoretsanitis et al. 2018), two of them additionally found a negative correlation between BMI and ODV, but not for VEN (Warrings et al. 2021; Schoretsanitis et al. 2018). Differences between groups did not exceed 50%.

#### Drug–drug interactions with comedication

A total of 14 studies discussed potentially interfering concomitant medication on VEN treatment, assessed as changes in dose-corrected BLs. Most of them were cross-sectional studies (see Table 2). Higher ODV levels (27% and 51%) were found in patients co-administered with the mood stabilizer valproate, a known inhibitor of CYP2C19, CYP2C9, and CYP3A4 metabolizing enzymes (Wang et al. 2020; Unterecker et al. 2014). Care has also to be taken in case of add-on therapies with other antidepressants. An increase of the active moiety (46%) and of VEN (232%) was for example reported after the add-on therapy with the antidepressant doxepin that has CYP2D6 inhibiting effects (Paulzen et al. 2018). An antidepressant combination with trimipramine was also found to considerably influence VEN (an increase of 53%) and active moiety (an increase of 38%) BLs (Kowalewski et al. 2019). In clinical practice, the antipsychotic agent Melperon is often prescribed on demand due to its sedative effect. Due to its strong CYP2D6 inhibiting effects, melperon was found to increase dose-corrected VEN BLs by 237% while decreasing ODV BLs by 54%. The MPR was 85% lower in patients co-administered with Melperon (Grözinger et al. 2003). Smaller influences on plasma levels (less than 50%) were found in additional treatments with lorazepam and clozapine (Wang et al. 2020; Grasmäder et al. 2004). The concomitant use of non-psychiatric medication might also pose a challenge in VEN treatment. Pharmacokinetic influences were described for the commonly co-prescribed omeprazole (34% higher active moiety BLs) (Kuzin et al. 2018), for pantoprazole (Kuzin et al. 2018), and for amlodipine (lower MPR) (Augustin et al. 2018). No effect on the BLs or MPR was found from pindolol, mirtazapine, lithium, and aripiprazole. Against expectations, one study did not find any differences in serum levels of patients receiving weak CYP2D6 inhibitors (Stamm et al. 2014). Possible explanations are the small number of patients being co-treated with CYP2D6 inhibitors (*n* = 11; 12,4%) and the weak inhibitory potential of the investigated substances (risperidone, olanzapine, atorvastatin, and simvastatin). In summary, several medications have been identified as factors influencing ODV exposure with a concurrent effect on the MPR.

#### Effects of CYP2D6 and CYP2C19 genotypes

Eight studies were identified that report an association between CYP2D6 or CYP2C19 metabolizer states and dose-corrected BLs, MPRs, or antidepressant efficacy (Lobello et al. 2010; Waade et al. 2014; Komahashi-Sasaki et al. 2020; Hermann et al. 2008; Kringen et al. 2020; Mannheimer et al. 2016; McAlpine et al. 2011; Ganesh et al. 2021). One study found CYP2D6 enzyme activity being positively associated with antidepressant response, but not with VEN or ODV BLs (Lobello et al. 2010). Five studies reported higher dose-corrected VEN BLs in genotypes with a lower CYP2D6 or combined CYP2D6/CYP2C19 activity (Waade et al. 2014; Hermann et al. 2008; Kringen et al. 2020; McAlpine et al. 2011; Ganesh et al. 2021). The differences between PMs and normal metabolizers (NM) ranged between 100 and 1242% (Waade et al. 2014; Hermann et al. 2008; Kringen et al. 2020; McAlpine et al. 2011). Accordingly, four studies reported a large decrease of dose-corrected ODV BLs in the respective PM patients by 62% up to the complete absence of ODV in the blood (Waade et al. 2014; Hermann et al. 2008; Kringen et al. 2020; McAlpine et al. 2011). As expected, the observed changes are also reflected in the MPR of VEN (ODV/VEN). Lower MPRs were found in patients with CYP2D6 PM status when compared to the NM group in six studies (Waade et al. 2014; Komahashi-Sasaki et al. 2020; Hermann et al. 2008; Mannheimer et al. 2016; McAlpine et al. 2011; Ganesh et al. 2021). The decrease of ODV/VEN in PMs reached up to 1400%, starting at 79% (Waade et al. 2014; Hermann et al. 2008; Mannheimer et al. 2016). Dose-corrected AM BLs were reported to be higher in CYP2D6 PMs or combined CYP2D6/CYP2C19 PMs than in NMs in six studies (Waade et al. 2014; Komahashi-Sasaki et al. 2020; Hermann et al. 2008; Kringen et al. 2020; McAlpine et al. 2011; Ganesh et al. 2021), reaching significance in four of them (Waade et al. 2014; Kringen et al. 2020; McAlpine et al. 2011; Ganesh et al. 2021). The differences were consistently above 50% and reached up to 260%. Only one study showed a clinically non-relevant decrease of 23% in AM BLs in CYP2D6 PMs under the age of 40 compared to NMs (Waade et al. 2014).

## Discussion

The present work systematically explores concentration-efficacy assumptions for the antidepressant drug VEN following a guideline-like methodology. This is the first quantitative analysis, supporting higher active moiety BLs in responders compared to non-responders to VEN treatment, but not for VEN or ODV alone. The qualitative evaluation still finds a limited grade of evidence for a relationship between antidepressant effect and drug concentration (level C, low). This also holds true for the occurrence of ADRs (level C, low for tremor).

### Therapeutic reference range for venlafaxine

The relationships of BLs of VEN, its active metabolite, and the active moiety have been shown to be linear with applied VEN doses. ODV constitutes 62% of the active moiety (MPR: 223 ng/ml / 358 ng/ml = 0.623; *k* = 11, *n* = 3,200). After the administration of approved doses in (severe) depression (ER 75–375 mg/day), expected active moiety and ODV BLs range from 140 to 701 ng/ml and 85–424 ng/ml (Table 3). Interquartile ranges in study patients treated under flexible dosing were somewhat higher than expected active moiety and ODV BLs (140–421 ng/ml and 85–254 ng/ml) after the administration of recommended maintenance doses in depression (ER 75–225 mg/day) (*n* = 3200): 225–450 ng/ml and 144–302 ng/ml. The IQR of responders (*n* = 82; 50% reduction in HAM-D scores) after 8 weeks of treatment with VEN for depression was 305–534 ng/ml (AM) and 213–382 ng/ml (ODV) (Engelmann et al. 2021). The corresponding ROC analysis revealed a threshold ODV concentration of 289 ng/ml for antidepressant response (Engelmann et al. 2021). De Donatis and colleagues reported a u-shaped active moiety concentration/effect relationship with optimal efficacy within 100–400 ng/ml, referring to a range between the onset (30%) and maximum (42%) reduction in HAMD-21 score after 3 months of treatment (Eichentopf et al. 2022). Patients with active moiety concentrations above 400 ng/ml were more often found to develop a tremor compared to patients within the current reference range of 100–400 ng/ml (Engelmann et al. 2021). One PET study reports SERT occupancy in relation to ODV BLs (Frankle et al. 2018). A total of 80% SERT occupancy is reached above 85 ng/ml. Based on our results, we suggest a target range of 85–380 ng/ml for ODVs’ antidepressant efficacy. The lower level hereby indicates an expected concentration from the lowest dose (75 mg/day) recommended for maintenance therapy in real-world patients. This is supported by SERT occupancy findings (EC_80_) from one neuroimaging study (Frankle et al. 2018). The suggested upper level of ODV’s efficacy range of 380 ng/ml is based on the 75th interquartile concentration in responders and reflects a therapeutic ceiling. Thus, increased occurrences of side effects, in particular tremor, are expected at higher drug concentrations. Over 75% of all patients included in our meta-analysis showed drug concentrations below the upper threshold. Based on the determined threshold ODV concentration of 289 ng/ml for antidepressant response and the previously calculated ODV percentage of the active moiety (62%), we further suggest a target range of 140–600 ng/ml for the active moiety. This represents a pharmacokinetically-expected concentration range (MPR 0.6, *n* = 2,751).

### Rationale for the use of TDM in venlafaxine

For VEN, the 25th interquartile concentrations of patients (144 ng/ml) and of responders (213 ng/ml) to the drug treatment are quite high compared to the SERT occupancy threshold. However, some patients might benefit already from low concentrations, and some might require additional NET actions at higher drug concentrations to reach optimal antidepressant efficacy. In contrast to other psychotropic drugs, a dose titration towards higher doses within the proposed reference range is indicated for VEN in case of insufficient response with BLs within the lower to medium part of the reference range. Even at high doses/concentrations, the incidence of ADRs in VEN-treated patients was generally low. A correlation between insufficient metabolization (CYP2D6) of VEN to ODV and the occurrence of several ADRs was found in some studies. A dose of 150 mg/day was suggested as a cutoff for the onset of NET effects in previous studies (Eap et al. 2021). According to our findings, this would correspond to a minimum level of 170 ng/ml for ODV and 280 ng/ml for the active moiety, right within the target range proposed in this study. Sex, age, certain CYP-inhibiting comedications, and CYP2D6/CYP2C9 metabolizer status were identified as clinically relevant factors on VEN, ODV, and active moiety BLs. Dose-related concentrations (C/D ratios) strongly varied among different trials (data supplement, table 10, 11). As shown for the antipsychotic drug aripiprazole in a similar study (Hart et al. 2022a), patients who are co-medicated with CYP2D6 inhibitors, but also CYP2C19 inhibitors, or that are CYP2D6 or CYP2C19 PM will show increased VEN levels with constant to decreased ODV levels. This will also impact the respective MPRs and most plausibly the effectiveness of VEN treatment. Consequently, a dose titration under consideration of the active moiety alone is not sufficient. Measurement of ODV is obligatory to assess the metabolization of VEN to its active metabolite. Relying on the sole active moiety BL could lead to improper overdosing of potentially poor metabolizers and increased occurrence of ADRs. CYP2D6 polymorphisms have furthermore been shown as ethnicity-related (Teh and Bertilsson 2012). As a result, in clinical practice, both, the ODV and the active moiety blood levels should be measured and evaluated in regard to the suggested reference ranges. TDM is particularly indicated for patients who have been newly prescribed with VEN due to a variety of powerful factors that can affect blood levels. The strong positive correlation between BLs and age further highlights the importance of TDM in the elderly (> 65 years).

### Limitations

Limitations of the suggested reference ranges refer to the quality of the underlying study design that highly varies among psychotropic drug trials. As in similar studies on therapeutic reference ranges for other psychotropic drugs before (Eichentopf et al. 2022; Hart et al. 2022b), the presented information was mostly extracted from naturalistic TDM studies or small non-controlled studies. High-quality randomized controlled trials investigating concentration/efficacy relationships and studies using a placebo lead-in phase are missing to support a target range for VEN. Low-quality study design might be the reason for reporting artificial concentration/effect relationships (Schoretsanitis et al. 2019; Veefkind et al. 2000). Flexible dose design was identified as an influencing factor leading to artificial findings (Funk et al. 2022). Other reasons may be the poor consideration of potential drug–drug interactions and heterogeneous reporting of VEN or ODV BLs.

Despite findings of a good correlation between serum and brain concentrations (Paulzen et al. 2015), only weak assumptions can be drawn from neuroimaging studies due to limitations in published data such as missing EC_50_ values and differing affinities of VEN/ODV to SERT and NET among studies. An active moiety threshold of 400 ng/ml would relate to a NET occupancy of 37%. A clear cut-off relating to antidepressant efficacy of NET has to be defined by future studies. The primary focus of this study was to provide a full overview of the efficacy and safety of VEN with regard to optimal target ranges. We did not consider dose/response studies that might have provided additional insights. Former guidelines (Hiemke et al. 2018) take data on prolongation of corrected QT-(QTc)-time into account when determining target ranges for VEN. Only one study was identified assessing the correlation between active moiety BL and QTc-time prolongation (Hefner et al. 2019). However, only three of 27 patients in this sample showed critical QTc-times of over 450 ms with two of them having BLs above 400 ng/ml. Studies investigating the incidence of QT prolongation/ADR might in the future be considered in this regard.

## Conclusion

Based on our results, we suggest a target range of 85– 380 ng/ml for ODV and 140–600 ng/ml for the active moiety of VEN for the treatment of depressive disorders. There is a chance of poor response at subtherapeutic concentrations and TDM within the suggested therapeutic reference range will increase the probability of response. A titration towards higher doses within the proposed reference range is recommended in case of non-response in the lower to medium part of the reference range. The risk for ADRs increases with drug titration towards higher concentrations, but in particular with supratherapeutic drug concentrations. Some patients will benefit at low doses predominantly from the serotonergic effects of VEN. According to the findings of the included studies, these might be patients, that suffer from a combination of anxiety and depressive disorder, while the anxiety component is somewhat stronger. Some patients, however, might require higher doses to achieve sufficient norepinephrine transporter occupancy. This goes in line with the recommendation of lower VEN doses for the treatment of patients with anxiety or social phobia and higher VEN doses for the treatment of depressive disorders. As a result, dose titration is strongly recommended in case of non-response at the lower range of the recommended therapeutic reference range. In clinical practice, TDM can be a valuable tool to guide dose adjustments towards, but also within the therapeutic reference range for VEN to reach optimal antidepressant response.

### Supplementary information

ESM 1 S1. Table Inclusion and exclusion criteria for study eligibility. S2. Full database search strings. S3. PRISMA flow diagram. S4. Figure a Risk of bias eligible RCTs. Figure b Summary of risk of bias assessment. S5. Table quality Assessment of the therapeutic drug monitoring component for all studies. S6. Table study type specific quality assessment for cohort studies. S7. Table study type specific quality assessment for cross-sectional studies. S8. Study details concentration/effect studies. S9. Study details concentration studies. S10. Study details neuroimaging studies. S11. Table findings from neuroimaging studies. S12. Abbreviations used in the data supplement. (DOCX 520 kb)

## Data Availability

The datasets generated during and/or analyzed during the current study are available from the corresponding author upon reasonable request.
