# Supplementary material for: Venlafaxine’s therapeutic reference range in the treatment of depression revised: a systematic review and meta-analysis
Source: Psychopharmacology (Berl). 2023 Oct 19;241(2):275–89. doi: 10.1007/s00213-023-06484-7 (PMC10806172; doi:10.1007/s00213-023-06484-7)
Supplement: Supplementary file 1 — S1. Table Inclusion and exclusion criteria for study eligibility. S2. Full database search strings. S3. PRISMA flow diagram. S4. Figure a Risk of bias eligible RCTs. Figure b Summary of risk of bias assessment. S5. Table quality Assessment of the therapeutic drug monitoring component for all studies. S6. Table study type specific quality assessment for cohort studies. S7. Table study type specific quality assessment for cross-sectional studies. S8. Study details concentration/effect studies. S9. Study details concentration studies. S10. Study details neuroimaging studies. S11. Table findings from neuroimaging studies. S12. Abbreviations used in the data supplement. (DOCX 520 kb) [file 213_2023_6484_MOESM1_ESM.docx]

Data Supplement

“Venlafaxine’s therapeutic reference range revised: A systematic review and meta-analysis”

X.M. Lense^1^, C. Hiemke^2,10^, C.S.M. Funk^3^, U. Havemann-Reinecke^4,10^, G. Hefner^5,10^, A. Menke^6,10,11^, R. Mössner^7,10^, T.G. Riemer^3^, M. Scherf-Clavel^8,10^, G. Schoretsanitis^9,10^, G. Gründer^1,10^, X.M. Hart^1,10^

*^1^Central Institute of Mental Health, Department of Molecular Neuroimaging, University of Heidelberg, Mannheim.*

*^2^University of Mainz, Clinic of Psychiatry and Psychotherapy.*

*^3^Charité University, Institute of Clinical Pharmacology and Toxicology, Berlin.*

*^4^University of Göttingen, Clinic of Psychiatry and Psychotherapy.*

*^5^Vitos Clinic of Forensic Psychiatry, Eltville.*

*^6^Psychosomatic Clinic Medical Park Chiemseeblick, Bernau a. Chiemsee.*

*^7^University Hospital of Tübingen, Department of Psychiatry and Psychotherapy.*

*^8^University Hospital of Würzburg, Department of Psychiatry, Psychosomatics and Psychotherapy. ^9^University Hospital of Zürich, Department of Psychiatry, Psychotherapy and Psychosomatics, Zürich, Switzerland.*

*^10^Arbeitsgemeinschaft für Neuropsychopharmakologie und Pharmakopsychiatrie (AGNP), Working Group „Therapeutic Drug Monitoring“*

*^11^ Department of Psychiatry and Psychotherapy, University Hospital, Ludwig Maximilian University of Munich,* *Munich, Germany.*

Correspondence:

[Xenija.Lense@zi-mannheim.de](mailto:Xenija.Lense@zi-mannheim.de)

*S1. Table inclusion and exclusion criteria for study eligibility.*

| Inclusion criteria all studies | Exclusion criteria all studies |
| --- | --- |
| - Human subjects - The study concerns VEN - Oral VEN psychotropic monotherapy arm or period of observation (at least one VEN blood level measurement before add-on therapy) | - Not written in English or German - No VEN blood levels or C/D ratios reported - No abstract available - Studies primarily comparing blood analysis techniques - Data from simulation studies - Papers containing the same data - Case reports & case series - Grey literature - Reviews & experts’ opinions - Postmortem studies - Maternal use during pregnancy or lactation |
| Additional inclusion criteria | **Additional exclusion criteria** |
| *a. concentration/effect studies*   - Direct clinical outcome measures are reported, i.e., safety or efficacy using a standardized rating scale (e.g., HAMD, MADRS, CGI)* | 1. *concentration/effect studies*  - VEN blood level is not measured in the steady state - Studies investigating drug effects in healthy volunteers |
| *b. concentration studies*   - Studies investigating blood levels in relation to dose in the steady state (3 days) | 1. *concentration studies*  - VEN blood level is not measured in the steady state (3 days) - Studies investigating pharmacokinetics in healthy volunteers |
| *c. neuroimaging studies*   - Studies investigating VEN blood concentrations in relation to SERT occupancy - Single dose studies - Studies investigating VEN concentrations in healthy volunteers |  |
| *d. Reviews & meta-analysis investigating a concentration/effect relationship for VEN* |  |

*Biomarkers (e.g. QTc-time) are not regarded a direct clinical outcome measure.

*S2. Full database search strings.*

| *PubMed* |
| --- |
| ("Venlafaxine Hydrochloride"[MeSH Terms] OR "Venlafaxine"[tw] OR "venlafaxine hydrochlorid*"[tw] OR "Trevilor"[tw] OR "Effexor"[tw]) AND ("serum level*"[tw] OR "plasma level*"[tw] OR "blood level*"[tw] OR "drug level*"[tw] OR "serum concentration*"[tw] OR "plasma concentration*"[tw] OR "blood concentration*"[tw] OR "drug concentration*"[tw] OR "Drug Monitoring"[Mesh] OR "drug monitor*"[tw] OR “positron emission tomography”[MeSH Terms] OR "Positron Emission Tomogra*"[tw] OR "PET scan*"[tw] OR "Tomography, Emission Computed, Single Photon"[Mesh] OR "Single Photon Emission*"[tw] OR "SPECT"[tw] OR "CAT Scan"[tw] OR "single photon emission computed tomography computed tomography"[MeSH Terms]) NOT ("Animals"[MeSH Terms] NOT "humans"[MeSH Terms]) |
| *Web of Science Core Collection* |
| (TS=Venlafaxine OR TS=Venlafaxine Hydrochloride OR TS=Trevilor OR TS=Effexor) AND (TS =(serum NEAR/1 level*) OR TS = (plasma NEAR/1 level*) OR TS= (blood NEAR/1 level*) OR TS=(drug NEAR/1 level*) OR TS=(serum NEAR/1 concentration*) OR TS=(plasma NEAR/1 concentration*) OR TS=(blood NEAR/1 concentration*) OR TS=(drug NEAR/1 concentration*) OR TS=(drug NEAR/1 monitor*) OR TS=(positron NEAR/1 emission NEAR/1 tomogra*) OR TS=(PET NEAR/1 scan*) OR TS=(single NEAR/1 photon NEAR/1 emission*) OR TS=SPECT OR TS=(CAT NEAR/1 Scan)) |
| *Cochrane Library* |
| (((MA Venlafaxine Hydrochloride) OR ("venlafaxin*" OR "Venlafaxine" OR "venlafaxine hydrochlorid*" OR "Trevilor" OR "Effexor")):ti,ab,kw) AND ([mh "positron emission tomography"] OR [mh "Tomography, Emission-Computed, Single-Photon"] OR [mh "single photon emission computed tomography computed tomography"] OR (positron NEAR/1 emission NEAR/1 tomogra* ) OR (PET NEAR/1 scan*) OR (tomography, emission NEAR/1 computed, single NEAR/1 photon) OR (single NEAR/1 photon NEAR/1 emission*) OR SPECT OR (CAT NEAR/1 Scan) OR (single NEAR/1 photon NEAR/1 emission) OR (single NEAR/1 photon NEAR/1 emission NEAR/1 computed NEAR/1 tomography NEAR/1 computed NEAR/1 tomograph*):ti,ab,kw OR (drug NEAR/1 monitor*):ti,ab,kw OR (serum NEAR/1 level*) OR (plasma NEAR/1 level*) OR (blood NEAR/1 level*) OR (drug NEAR/1 level*) OR (serum NEAR/1 concentration*) OR (plasma NEAR/1 concentration*) OR (blood NEAR/1 concentration*) OR (drug NEAR/1 concentration*)):ti,ab,kw |
| *PsycINFO* |
| (MA "Venlfaxine Hydrochloride" OR "venlfaxine" OR "venlafaxine hydrochloride" OR "Trevilor" OR "Effexor") AND (MA "positron emission tomography" OR "positron emission tomogra*" OR "pet scan*" OR MA "tomography, emission computed, single photon" OR "single photon emission*" OR "SPECT" OR "CAT Scan" OR MA "single photon emission computed tomography computed tomography" OR MA "Drug Monitoring" OR "Drug Monitoring" OR "serum level*" OR "plasma level*" OR "blood level*" OR “drug level*" OR "serum concentration*" OR "plasma concentration*" OR "blood concentration*" OR “drug concentration*") NOT (MA "Animals" NOT MA "humans") |

*S3. PRISMA flow diagram.*


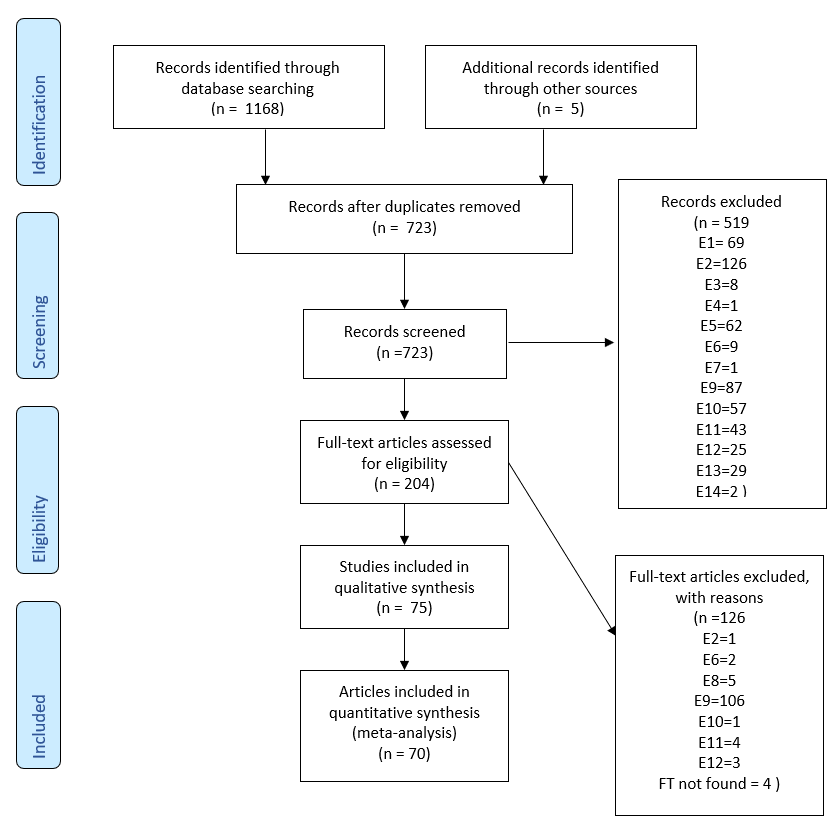


*S4. Figure a Risk of bias eligible RCTs. Figure b Summary of risk of bias assessment.*


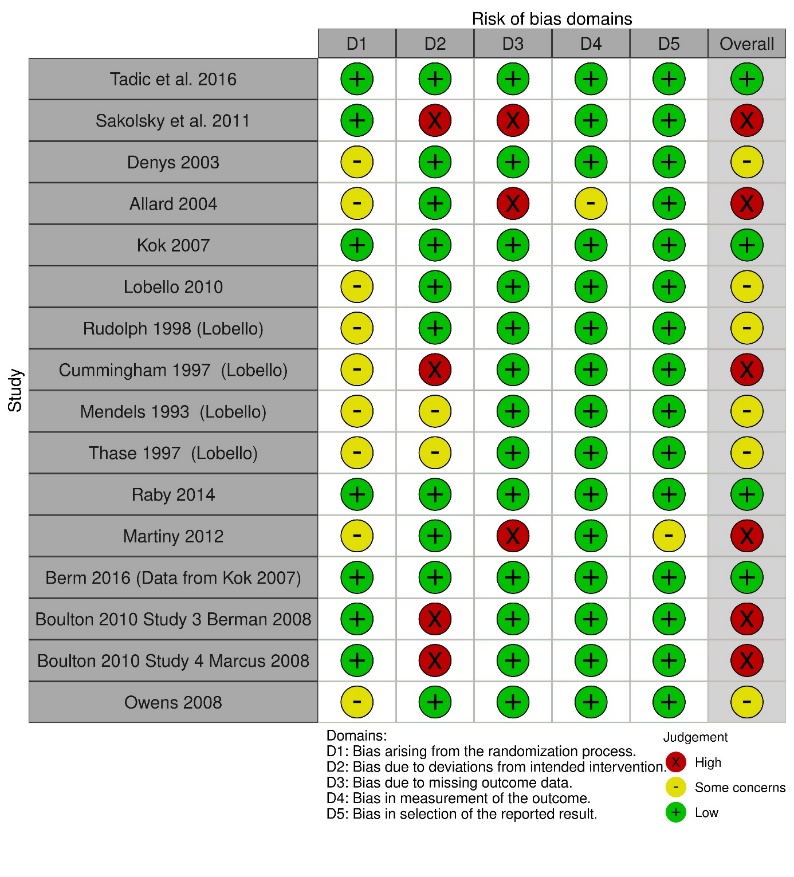


*Plot created with Risk-of-bias Visualization package (McGuinness, L. A., & Higgins, J. P. T. (2020). Risk-of-bias VISualization (robvis): An R package and Shiny web app for visualizing risk-of-bias assessments. Research Synthesis Methods, n/a(n/a). doi:10.1002/jrsm.1411)*

a.

b.


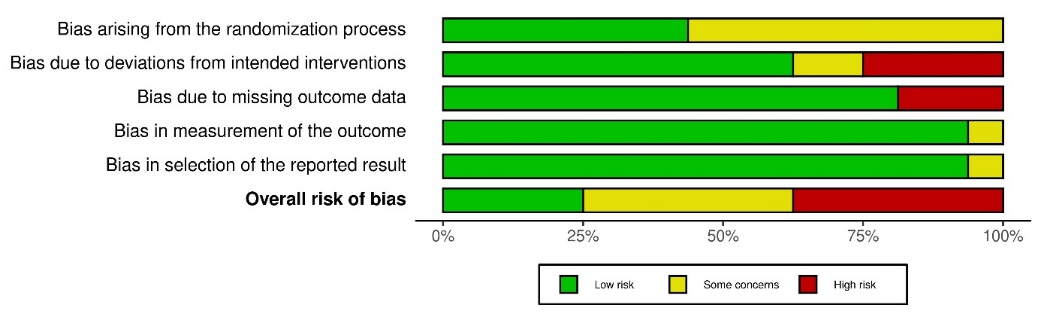


*Plot created with Risk-of-bias Visualization package (McGuinness, L. A., & Higgins, J. P. T. (2020). Risk-of-bias VISualization (robvis): An R package and Shiny web app for visualizing risk-of-bias assessments. Research Synthesis Methods, n/a(n/a). doi:10.1002/jrsm.1411)*

*S5. Table quality assessment of the therapeutic drug monitoring component for all studies.^1^*

| **Reference** | **Selection** | | **Comparability** | | **Drug Monitoring** | | | **Final Score** |
| --- | --- | --- | --- | --- | --- | --- | --- | --- |
|  | **Q1** | **Q2** | **Q3** | **Q4** | **Q5** | **Q6** | **Q7** |  |
| 1. ***concentration/effect studies*** | | | | | | | | |
| **Cohort studies** |  |  |  |  |  |  |  |  |
| *Cristancho et al. 2017* | o | xx | o | o | x | ox | ox | 5/10 |
| *Charlier et al. 2000* | x | xx | ? | o | o | xo | ox | 5/10 |
| *Charlier et al. 2002 Study 1* | x | xx | x | o | x | xx | ox | 8/10 |
| *Charlier et al. 2002 Study 2* | x | xx | x | o | x | xx | xo | 8/10 |
| *Ciusani et al. 2004* | x | xx | o | o | x | xx | xx | 8/10 |
| *Gex-Fabry et al. 2004 (D1)* | x | xx | o | x | x | xx | xx | 9/10 |
| *Grasmäder et al. 2004* | x | xo | o | ? | x | xx | xo | 6/10 |
| *Hoencamp et al. 2000* | o | xx | o | x | x | xx | xx | 8/10 |
| *Ozbey et al. 2017* | x | xx | o | o | x | xx | o? | 6/10 |
| *Scherf-Clavel et al. 2020* | x | xo | o | o | x | xx | ox | 6/10 |
| *Findling et al. 2007* | o | xx | o | o | x | xx | xx | 7/10 |
| *Stamm et al. 2014* | x | xx | o | x | o | xx | ox | 7/10 |
| *Shams et al. 2006* | x | xx | o | ? | x | ox | xx | 7/10 |
| *Whyte et al. 2006* | o | xx | o | o | x | xx | x? | 6/10 |
| *Proft et al. 2014* | x | xo | o | o | x | xx | ox | 7/10 |
| *Steen et al. 2015* | x | xo | o | ? | x | oo | ox | 4/10 |
| *De Donatis et al. 2021* | x | xx | x | o | x | xx | ox | 8/10 |
| **Crosssectional studies** |  |  |  |  |  |  |  |  |
| *Lloret-Linares et al. 2018* | x | xo | o | o | o | xx | ox | 5/10 |
| *Schoretsanitis et al. 2019 (D2)* | o | xo | o | o | x | xx | ox | 5/10 |
| *Schoretsanitis et al. 2019 (ADRs) (D2)* | x | ?o | o | o | x | xx | ox | 6/10 |
| **Randomized controlled trials** |  |  |  |  |  |  |  |  |
| *Allard et al. 2004* | o | xx | o | o | ? | xo | xx | 5/10 |
| *Denys et al. 2003* | x | xx | o | x | x | x? | xx | 8/10 |
| *Kok et al. 2007 and Berm et al. 2016* | o | xx | o | o | ? | x? | xx | 5/10 |
| *Lobello et al. 2010* | x | xx | x | o | ? | xo | ?x | 6/10 |
| *Martiny et al. 2012* | x | xx | o | x | o | xx | xx | 8/10 |
| *Raby et al. 2014* | o | xx | ? | o | ? | x? | xx | 5/10 |
| *Sakolsky et al. 2011* | o | xx | ? | o | x | xx | xx | 7/10 |
| *Tadic et al. 2016 + Engelmann et al. 2021* | o | xx | o | o | x | xx | xx | 7/10 |
| *Owens et al. 2008* | x | xx | o | x | o | x? | xx | 7/10 |
| *Aldosary et al. 2021* | x | xx | o | o | ? | xx | xx | 7/10 |
| 1. ***concentration studies*** | | | | | | | | |
| **Cohort studies** | | | | | | | | |
| *Gex-Fabry et al. 2002 (D1)* | x | xx | o | x | x | xx | xx | 9/10 |
| *Grözinger et al. 2003* | x | ?? | o | o | x | xx | o? | 4/10 |
| *Hefner et al. 2015* | x | ?? | x | o | x | xx | x? | 6/10 |
| *Silhan et al. 2018* | x | xo | o | ? | x | xx | xx | 7/10 |
| *Tveit et al. 2020* | x | ?? | ? | ? | x | xo | oo | 3/10 |
| **Crosssectional studies** | | | | | | | | |
| *Augustin et al. 2018* | x | ?? | x | o | x | xx | ox | 6/10 |
| *Chermá et al. 2008* | o | ?? | o | o | x | xx | oo | 3/10 |
| *Fekete et al. 2020* | x | ?o | x | o | x | xx | ox | 6/10 |
| *Hansen et al. 2017* | o | ?? | ? | ? | x | x? | ox | 3/10 |
| *Hermann et al. 2008* | x | ?? | o | o | x | xx | o? | 4/10 |
| *Jukic et al. 2021* | x | ?? | ? | ? | x | xo | o? | 3/10 |
| *Komahashi-Sasaki et al. 2020 + Sasaki et al.2021* | o | xo | o | o | x | xx | ox | 5/10 |
| *Kowalewski et al. 2019 (D2)* | x | oo | x | o | x | xx | ox | 6/10 |
| *Kringen et al. 2020* | x | ?? | ? | o | x | xx | ox | 5/10 |
| *Kuzin et al. 2018 (D2)* | x | ?? | x | o | x | oo | ox | 6/10 |
| *Mannheimer et al. 2016* | x | ?? | ? | ? | x | xx | o? | 4/10 |
| *Mc Alpine et al. 2011* | x | ?? | o | o | x | xx | o? | 4/10 |
| *Paulzen et al. 2015* | x | xx | ? | o | x | xo | ox | 6/10 |
| *Paulzen et al. 2018 (D2)* | x | ?? | x | o | x | ?? | ox | 4/10 |
| *Reis et al. 2009* | x | ?? | o | ? | x | ?? | ox | 3/10 |
| *Reis et al. 2002* | x | ?? | ? | o | x | xx | ox | 5/10 |
| *Schoretsanitis et al. 2018 (D2)* | x | ?o | x | ? | x | ?? | ox | 4/10 |
| *Scherf-Clavel et al. 2019* | x | ?o | ? | ? | x | xx | ox | 5/10 |
| *Sigurdsson et al. 2014* | x | xo | x | ? | x | xx | ox | 7/10 |
| *Unterecker et al. 2011* | x | ?? | ? | ? | o | ?x | ox | 3/10 |
| *Unterecker et al. 2012* | x | ?o | o | o | x | ?x | ox | 4/10 |
| *Unterecker et al. 2014a* | o | ?? | ? | o | x | ?? | ox | 2/10 |
| *Unterecker et al. 2014b* | x | ?? | x | o | o | ?? | ox | 3/10 |
| *Waade et al. 2014* | o | ?? | ? | o | x | xx | ox | 4/10 |
| *van der Weide et al. 2005* | o | ?? | ? | o | x | xx | ?? | 3/10 |
| *Wang et al. 2020* | o | xo | ? | o | x | xx | ox | 5/10 |
| *Warrings et al. 2021* | x | ?? | ? | o | x | xx | ox | 5/10 |
| *Ganesh et al. 2021* | x | ?? | o | ? | x | xo | o? | 3/10 |
| **Randomized controlled trials** | | | | | | | | |
| *Berman et al. 2007 + Marcus et al. 2008 (study 3 + 4)* | x | xx | o | o | o | xo | x? | 5/10 |
| 1. ***neuroimaging studies*** | | | | | | | | |
| **Cohort studies** | | | | | | | | |
| *Arawaka et al. 2019* | x | xx | o | x | o | xo | xx | 8/10 |
| *Frankle et al. 2017* | o | ox | x | x | ? | ox | xx | 6/10 |
| *Meyer et al. 2004* | o | ox | x | o | o | xx | ox | 5/10 |
| *Shang et al. 2007* | o | xx | x | x | x | xx | xo | 8/10 |

^1^Due to a very short elimination half-life, steady state for the active moiety is presumed after three days of a constant oral dosing. Sampling should rather reflect a minimum than a maximum concentration in the blood, optimally a time point immediately prior to the next dose. Comedication with CYP2D6, CYP2C19, or CYP3A4 inducers or inhibitors potentially alters pharmacokinetics in the study sample, resulting in a variation in metabolite-to-parent compound ratios (MPRs). Comedication might plausibly influence venlafaxine pharmacodynamics and thus was covered by quality assessment. Changes in drug efficacy among CYP genetic polymorphism groups with altered MPRs support this statement (Shams, M. E. E., Arneth, B., Hiemke, C., Dragicevic, A., Muller, M. J., Kaiser, R., . . . Hartter, S. (2006). CYP2D6 polymorphism and clinical effect of the antidepressant venlafaxine. Journal of Clinical Pharmacy and Therapeutics, 31(5), 493-502. doi:10.1111/j.1365-2710.2006.00763.x, Whyte, E. M., Romkes, M., Mulsant, B. H., Kirshne, M. A., Begley, A. E., Reynolds, C. F., 3rd, & Pollock, B. G. (2006). CYP2D6 genotype and venlafaxine-XR concentrations in depressed elderly. Int J Geriatr Psychiatry, 21(6), 542-549. doi:10.1002/gps.1522)

Furthermore, polymorphisms in CYP2D6 have been shown to be ethnicity related. BL measurements in clinical studies should be performed using a validated method and cover concentrations of 10% around the currently defined lower threshold.

*S6. Table study type specific quality assessment for cohort studies.^2^*

| **Reference** | **Selection** | | | | **Comparability** | **Outcome** | | | | **Final score** |
| --- | --- | --- | --- | --- | --- | --- | --- | --- | --- | --- |
|  | **Q1** | **Q2** | **Q3** | **Q4** | **Q5** | **Q6** | **Q7** | **Q8** | **Q9** |  |
| 1. ***concentration studies*** | | | | | | | | | | |
| *Gex-Fabry et al. 2002 (D1)* | x | o | x | o | xx | x | x | x | x | 8/10 |
| *Grözinger et al. 2003* | x | o | ? | x | xo | x | x | x | x | 7/10 |
| *Hefner et al. 2015* | x | x | o | x | xx | x | x | x | x | 9/10 |
| *Silhan et al. 2018* | x | x | x | x | ox | x | x | x | x | 9/10 |
| *Tveit et al. 2020* | x | x | o | x | ox | x | x | o | x | 7/10 |
| 1. ***concentration/effect studies*** | | | | | | | | | | |
| *Cristancho et al. 2017* | o | ? | o | x | xx | x | x | x | x | 7/10 |
| *Charlier et al. 2000* | x | x | o | x | ox | o | x | x | x | 7/10 |
| *Charlier et al. 2002 Study 1* | x | o | o | x | xo | o | x | x | x | 6/10 |
| *Charlier et al. 2002 Study 2* | x | o | o | x | xo | o | x | x | x | 6/10 |
| *Ciusani et al. 2004* | x | o | x | x | oo | ? | x | x | x | 6/10 |
| *Gex-Fabry et al. 2004 (D1)* | x | ? | o | x | ox | ? | x | x | x | 6/10 |
| *Grasmäder et al. 2004* | x | x | o | x | oo | x | x | x | x | 7/10 |
| *Hoencamp et al. 2000* | o | o | o | x | oo | o | x | x | x | 4/10 |
| *Ozbey et al. 2017* | x | o | o | x | ox | o | x | o | x | 5/10 |
| *Scherf-Clavel et al. 2020* | x | ? | o | x | oo | x | x | o | x | 5/10 |
| *Findling et al. 2007* | o | o | x | x | xo | o | x | x | x | 6/10 |
| *Stamm et al. 2014* | x | ? | o | x | xx | x | x | o | x | 7/10 |
| *Shams et al. 2006* | x | x | x | o | ox | o | x | o | x | 6/10 |
| *Whyte et al. 2006* | o | x | x | x | xx | o | x | x | x | 8/10 |
| *Proft et al. 2014* | x | o | o | o | xo | o | x | x | x | 5/10 |
| *Steen et al. 2015* | x | x | o | o | xo | x | x | x | x | 7/10 |
| *De Donatis et al. 2021* | x | o | x | x | xx | x | x | x | x | 9/10 |
| 1. ***neuroimaging studies*** | | | | | | | | | | |
| *Arawaka et al. 2019* | x | x | o | o | xx | x | x | x | o | 7/10 |
| *Frankle et al. 2017* | o | x | ? | x | xx | x | x | x | o | 7/10 |
| *Meyer et al. 2004* | o | x | ? | x | xo | x | x | x | o | 6/10 |
| *Shang et al. 2007* | o | o | x | x | xx | x | x | x | x | 8/10 |

*S7. Table study type specific quality assessment for cross-sectional studies.^2^*

| **Reference** | **Selection** | | | | **Comparability** | **Outcome** | | **Final score** |
| --- | --- | --- | --- | --- | --- | --- | --- | --- |
|  | **Q1** | **Q2** | **Q3** | **Q4** | **Q5** | **Q6** | **Q7** |  |
| 1. ***concentration studies*** | | | | | | | | |
| *Augustin et al. 2018* | x | o | o | o | xx | x | o | 4/8 |
| *Chermá et al. 2008* | o | o | x | o | oo | x | x | 3/8 |
| *Fekete et al. 2020* | x | o | x | o | xx | x | o | 5/8 |
| *Hansen et al. 2017* | o | o | o | o | ox | x | o | 2/8 |
| *Hermann et al. 2008* | x | o | x | o | ox | x | x | 5/8 |
| *Jukic et al. 2021* | x | o | ? | ? | oo | x | x | 3/8 |
| *Komahashi-Sasaki et al. 2020 and Sasaki et al. 2021* | o | o | x | o | ox | x | x | 4/8 |
| *Kowalewski et al. 2019 (D2)* | x | o | o | ? | xx | x | x | 5/8 |
| *Kringen et al. 2020* | x | o | o | o | ox | x | x | 4/8 |
| *Kuzin et al. 2018 (D2)* | x | o | o | o | xo | x | x | 4/8 |
| *Mannheimer et al. 2016* | x | o | o | o | ox | x | x | 4/8 |
| *Mc Alpine et al. 2011* | x | o | o | ? | ox | x | x | 4/8 |
| *Paulzen et al. 2015* | x | o | x | ? | ox | x | x | 5/8 |
| *Paulzen et al. 2018 (D2)* | x | o | o | ? | ox | x | x | 4/8 |
| *Reis et al. 2009* | x | o | x | ? | ox | x | x | 5/8 |
| *Reis et al. 2002* | x | o | o | ? | oo | x | x | 3/8 |
| *Schoretsanitis et al. 2018 (D2)* | x | o | x | ? | xx | x | x | 6/8 |
| *Scherf-Clavel et al. 2019* | x | o | x | ? | xo | x | x | 5/8 |
| *Sigurdsson et al. 2014* | x | o | x | o | ox | x | x | 6/8 |
| *Unterecker et al. 2011* | x | o | o | ? | ox | x | x | 4/8 |
| *Unterecker et al. 2012* | x | o | x | ? | xo | x | x | 5/8 |
| *Unterecker et al. 2014* | o | o | x | ? | xo | x | o | 3/8 |
| *Unterecker et al. 2014 (VA interaction study)* | x | o | x | ? | xx | x | o | 5/8 |
| *Waade et al. 2014* | o | o | x | o | ox | x | x | 4/8 |
| *van der Weide et al. 2005* | o | o | ? | ? | xo | x | x | 3/8 |
| *Wang et al. 2020* | o | o | x | x | ox | x | x | 5/8 |
| *Warrings et al. 2021* | x | o | x | o | ox | x | x | 5/8 |
| *Ganesh et al. 2021* | x | o | x | o | ox | x | x | 5/8 |
| 1. ***concentration/effect studies*** | | | | | | | | |
| *Lloret-Linares et al. 2018* | x | o | x | x | oo | ? | x | 4/8 |
| *Schoretsanitis et al. 2019 (D2)* | o | o | o | ? | xo | o | x | 2/8 |
| *Schoretsanitis et al. 2019 (D2)* | x | o | x | ? | ox | o | x | 4/8 |

^2^Six articles that refer to three original datasets were summarized into three studies. Another eight articles refer to two original datasets but investigate different leading questions. They were marked with “D”. One article consists of two separately conducted studies and was included into the rating twice. Of note, some studies qualify their statement on performing drug measurement at trough level, when mentioning their limitations.

*S8. Study details concentration/effect studies ^3^.*

| **Authors, year** | **Study Design** | **Subjects**  **(% males) Indication**  **Mean ± SD Age** | **Country** | **Mean dose ± SD (range) (mg/d)** | **Mean VEN BL ± SD (ng/ml), if not specified other**  ***=converted from original** | **Mean ODV BL ± SD (ng/ml), if not specified other**  ***=converted from original** | **Mean AM BL ± SD (ng/ml), if not specified other**  ***=converted from original** | **Comment/**  **Main Outcome** |
| --- | --- | --- | --- | --- | --- | --- | --- | --- |
| **Cohort** | | | | | | | | |
| **Cristancho et al. 2017** | CS with flexible dose design | 233 (30%) *MDD* | Canada, US | 132.8 ± 48.2 | Non-TESI: 132.7 ± 133.6 TESI: 99.42 ± 109.2 | NA | NA | No C/E correlation found. |
| **Charlier et al. 2000** | CS with flexible dose design | Total: 119 VEN: 9  *MDD*  47.6 ± 12.7 | Belgium | **Responders** (n=7): 150  (75-225)  **Nonresponders** (n=2): 300  (225-375) | **Responders:**  Median: 44.23  2.5%-97.5%: 24.71 -400.29  **Nonresponders:** NA | NA | NA | No C/E correlation reported. |
| **Charlier et al. 2002 *Study 1*** | CS with flexible dose design | 76 *MDD* | Belgium | 193.4  (37.5-375.0) | **Responders:**  Median: 54  2.5%-97.5%: 10.13 - 200.00 | *Median: 139 | **Responders:**  197.7 ±79.9  Median: 193  2.5%-97.5%: 122.8–386.80  **Nonresponders:** 148.6 ±64.5 | No C/E correlation reported. |
| **Charlier et al. 2002 *Study 2*** | CS with flexible dose design | 22 (55%) *MDD* | Belgium | 75-300 | **Responders:**  195.38 ± 56.99 | NA | NA | Relationship between MADRS and PK could be found (p= 0,03), but not between CGI and PK. |
| **Ciusani et al. 2004** | CS with flexible dose design | 10 (30%) *Depressive Episode*  39 ± 8 | Switzerland | 195 ± 52  (75-225) f  lexible for the first 3 weeks, no dose adjustment in week 4 | [pg/ml per mg VEN] Day 28:  S-VEN: 278 ± 209 (35-751);  R-Ven: 313 ± 346  (12-1 153) | [pg/ml per mg VEN]  Day 28:  S-ODV: 559 ± 239 (232-1 072);  R-ODV: 532 ± 324  (21-1 151) | **Responders:**  203-359  **Nonresponders:** 172-564 | On day 28, responders had a significantly lower ODV/VEN ratio than non-responders. |
| **Gex-Fabry et al. 2004 (D1)** | CS with fixed dose design | 35 (40%) *moderate or severe depressive episode*  Median: 44 | Switzerland | 300  BID | **Nonresponders**:  192 (37–496)  **Nonresponders (transient responders)**:  160 (69–393)  **Persistent early responders**:  214 (74–1294)  **Persistent late responders**:  146 (27–320) | **Nonresponders:**  475 (298-569) **Nonresponders (transient):**  351 (285-734)  **Persistent early responders:**  589 (209-772)  **Persistent late responders**:  385 (175-708) | **Nonresponders:** 719 (466-819) **Nonresponders (transient):**  576 (418-803)  **Persistent early responders:**  725 (458-1605)  **Persistent late responders:**  554 (202-988) | No C/E association remained significant.  Study was not included in metaanalysis because of altered dose regimen. |
| **Grasmäder et al. 2004** | CS with flexible dose design | Total: 136 (43%) VEN: 15  *Affective Disorders (F3X)*  49 ± 14 | Germany | NA | NA | NA | C/D ratio:  0,3645 ng/ml/mg | No C/E found;  overall very low response rate: 28.6% (HAMD and CGI). |
| **Hoencamp et al. 2000** | CS with fixed dose design | Total: 60 (45%)  VEN: 37 (14 discontinued; ITT)  *MDD*  45 ± 12 | The Nether-lands | 75 (Week 1)  150 (Week 2-4) 225 (Week 5-7) | Week 1: 44  Week 4: 71  Week 7:114,3 | Week1: 98  Week 4: 161.85 Week 7: 224.07 | * Week1: 142  Week 4: 232.85 Week 7: 338.37 | Negative C/E found (in week 7 for VEN+ ODV) |
| **Ozbey et al. 2017** | CS  Dose design NA | 94  (41 dropouts) *MDD*  38.1±10.8 | Turkey | 81.5 ± 3.0 (37.5–150; median: 75) | Patients in LQ (< 12.27 ng/ml): n = 13  Patients with EC (12.27–19.96 ng/ml): n = 26  Patients in UQ (> 19.96 ng/ml): n = 13  No specific information on PK given. | NA | NA | No statistically significant C/E was observed. VEN-induced akathisia was more frequent in patients with the TT genotype for both the C3435T and G2677T/A polymorphisms. |
| **Scherf-Clavel et al. 2020** | CS with fixed dose design | 36 (47,2%), N=23 with TDM-data *multiple diagnosis*  35.9±13.4 | Germany | W4:  273.9 ± 74.5 | W4: 116.4 ± 88.3 | W4: 251.7 ± 96.8 | W4: 368.0 ± 123.4 | C/E found.  For remission, based on 21 patients with necessary information fully available, ROC analysis revealed that an association between the AM in week 4 and remission could be expected. |
| **Findling et al. 2007** | CS with flexible dose design | 38 (87%)  (4 patients discontinued) *ADHD*  10.7±3.5 | US | 1. Group: 0,5 mg/kg (max 37,5mg) 2. Group: 1.0 mg/kg (max. 75 mg) 3. Group: 2,0 mg/kg (max. 150 mg) | Day 8: 27.5 (15–60.5) (N=14) Day 15: 29.0 (14–92.5) (N=13) | Day 8: 84.0 (22–398) (N=26) Day 15: 84.8 (9.5–352) (N=26) | NA | No C/E reported.  Not included in metaanalysis, because of investigation of ADHD. |
| **Stamm et al. 2014** | CS with flexible dose design | Total: 204 after dropouts: 88 (29.5%)  *MDD*  45.39±13.02 | Germany | 225 mg/day from day 5 onward. | Mean serum level was 121.1 for venlafaxine (SD = 143.8) | 240.2 (SD = 94.6) | 361.3 (SD = 162.7) | C/E for ODV found, but not for VEN or AM. |
| **Shams et al. 2006** | CS with flexible dose design | Total: 100 (54%) Genotyping: 25 (56%)  *MDD, dysthymia, or depressive adjustment disorder*  52±11 | Germany | 183 ± 74  (range: 75-450) genotyped group: 215 ± 63 | Median: 93.5  (IQR: 50.2-183) Mean: 143.7 ± 138.8 (range: 14-6 530) | Median: 152  (IQR: 96.5-227.7) Mean: 171.3 ± 107.1 (19-499) | Median: 276  (IQR: 185-414.2) Mean: 315.8 ± 177.2 (53-954) | No C/E found.  Usage of IR formulation, therefore not included in our meta-analysis. |
| **Whyte et al. 2006** | CS with fixed dose design | Total: 46 (33%)  *MDD* 49.0±12.7 | US | WT:  142.5 ± 18.2 Median: 150 Range: 75-150  "mutant":  45.3 ± 12.8 Median: 150 Range:  112.5-150 | C/D WT: 0.69 ± 0.43 WT/*4 or *4/*4: 2.26 ± 2.80 | WT: 2.52 (1.27) WT/*4 or *4/*4: 1.74 ± 1.19 | NA | No C/E found.  VEN was significantly higher for those with WT/ *4 or *4/*4 genotype than those with two wild type alleles (WT/WT). ODV just the other way round. |
| **Proft et al. 2014** | CS  Dose design NA | 56 included patients (42.9%) *depressive episode due to MDD or BD*  41.57 ± 14.32 | Germany | 233.04 ± 75.58; (75–375) | NA | NA | 370.70 ± 181.84; (103–862) | C/E in 5-HTTLPR lAlA carriers, but not for the representative part of study population. |
| **Steen et al. 2015** | CS with flexible dose design | Total: 187 (44,9%)  VEN: 52  *SCZ*  41.57±14.32 | Norway | NA | NA | NA | Median: 170.91  IQR 309.42 and Median: 159.28 IQR: 207.20 | Positive C/E found.  Data not included because of the investigation of patients with SCZ. |
| **De Donatis et al. 2021** | CS with flexible dose design | 52 (44%)  *MDD*  55.73 ± 13.81 | Italy | 117.65 ± 40.40 | 83.84 ± 110.69 | 200.53 ± 159.28 | 284.39 ± 205.71  corrected for dosage:  75 mg (n=23):  239.87 ± 249.40  150 mg (n=27):  325.52 ± 157.95  225 mg (n=1):  198 | After excluding patients with more than 800 ng/mL and equal 0 ng/mL there was found a positive trend between BL and AR at month 1 and month 3.  Study was not included in meta-analysis, because response was defined as 30% HAMD reduction, which is not in line with other definitions. |
| **Crosssectional Studies** | | | | | | | | |
| **Lloret-Linares et al. 2018** | CSS with flexible dose design | Total: 67 (31,5%)  (3 dropouts) VEN: 7  *Multiple diagnosis*  49 | Switzerland | Median: 300 Range: 150-300 | Median: 20.02  Range: 0-112.6 | Median: 192.3  Range: 23.7-553.1 | Median: 204.8 Range: 23.7-665.7 | No C/E found. |
| **Schoretsanitis et al. 2019** | CSS with flexible dose design | Total 858 (41,7%)  *Multiple diagnosis* | Germany | Median (range) Resp: 225 (112.5-375) Nonresp.: 225 (112.5-475) | Median (range)  Resp: 79.5 (2.7–596.0) Nonresp.: 88.0  (1.6–3164.0) | Median (range)  Resp: 150.0  (3.2–505.0)  Nonresp.: 184.5  (0.6–1268.0) | Median (range) Resp.: 263.5  (5.9–1037.0)  Nonresp.: 309.5 (14.7–4432.0) | C/E only found after dichotomization 🡪 no C/E when PK and clinical outcome were investigated Disproportional distribution of responders and nonresponders (>85% nonresponse). |
| **Schoretsanitis et al. 2019** | CSS with flexible dose design | Total: 802  *Multiple diagnosis* | Germany | Median (range) ADR: 225  (75-450)  No ADR: 225 (37.5-475) | Median (range)  ADR: 75.0 (1.3-685.0)  No ADR:82.0 (1.6-3164.0) | Median (range)  ADR: 163.0  (13.0-635.0)  No ADR: 172.25  (0.6–1268.0) | Median (range) ADR: 265.5  (28.0-896.0)  No ADR: 284.5  (5.9-4432.0) | No C/E found. |
| **RCTs** | | | | | | | | |
| **Allard et al. 2004** | RCT with flexible dose design | Total: 148 (20%)  VEN: 73 (25%)  *MDD*  73.6 ± 5.9 | Sweden and Denmark | 1st Week:  VEN IR 37,5  2nd + 3rd Week: VEN ER 75  Nonresponders (n=41):  Week 4 + 5:  VEN ER 150 | * Week 8: 106,20 ± 139,83 Week 22: 55,32 ± 64,65 | * Week 8: 225,87 ± 192,47 Week 22: 161,87 ± 137,66 | * W8: 332,07 ± 237,90 W22: 217,19 ± 152,09 | No C/E found. |
| **Denys et al. 2003** | RCT with fixed dose design | Total: 150 VEN: 75(37%)  *OCD*  36 ± 12 | The Netherlands | Week 1: 75  W3: 150  W5: 225  W8: 300  W10: 300  W12: 300 | Week 1: 80 ± 108 W3: 133 ± 160 W5: 287 ± 343 W8: 360 ± 386 W10: 420 ± 434 W12: 336 ± 274 | Week 1: 123 ± 66  W3: 214 ± 86  W5: 305 ±149  W8: 356 ± 170  W10: 392 ± 195  W12: 356 ± 150 | * Week1:  203 ± 126,57;  W3: 347 ± 181,65; W5: 692 ± 373,97; W8: 716 ± 421,78; W10: 812 ± 475,80; W12: 692 ± 312,37 (händisch addierte Werte) | C/E found.  Not included in meta-analysis because of investigation of patients with OCD. |
| **Kok et al. 2007 and Berm et l. 2016** | RCT with flexible dose design and post hoc analysis | Total: 81 VEN: 40  (5 dropouts) Actual: 35  *MDD*  71.6 ± 6.8 | The Netherlands | 75 mg/day during the first 3 weeks. In case of insufficient response, the dose of venlafaxine ER was increased with 75 mg/day to a maximum of 375 mg/day | mean final plasma level: 430 (SD=278) | NA | NA | Berm et al.: negative correlation found after dichotomizing. |
| **Lobello et al. 2010** | Data from 4 short-term (6–12 weeks), randomized, double- blind, placebo-controlled studies (protocols 203, 208, 209, and 313)16–19 were pooled for analysis. | Total  (4 Studies): 836 (41,7)  VEN: 464 (38,8%)  *MDD*  EM:  40.62 ± 10.64  PM:  41.80 ± 10.70  PCB:  40.40 ± 11.16 | USA | Mean (SD), Median, Range EM:  129.08 ± 68.30, 129.75,  19.74-339.62 PM:  128.60 ± 60.47, 132.69,  22.97-316.35 | EM: 77.08 ± 8.54  PM: 276.76 ± 234.01 | EM: 221.37 ± 169.32 PM: 109.97 ± 94.55 | EM: 298.44 ± 232.72 PM: 386.73 ± 305.85 | No C/E found.  Differences between PMs and EMs: increased alkaline phosphatase, sweating, and insomnia. |
| **Martiny et al. 2012** | RCT with fixed dose design | Total: 31 VEN+PLC: 16 (68,7%)  *MDD*  45.3 ± 13.7 | Denmark | 150 mg from day 6 and for the rest of the study period. | Day 12 (n=13)  Mean: 171.75* SD: 134.90 Range: 26.87-521.05  Day 19 (n=12)  Mean: 162.60 SD: 137.95 Range: 26.04-503.88  **Responders:** 685 (827)* | Day 12  Mean: 224.47  SD: 104.74  Range: 24.21-371.59  Day 19  Mean: 201.32  SD: 90.53  Range: 25.53-321.58 **Responders:** 913 (375) nmol* | **Responders**  1599 (575) nmol* Week1: 203 ± 126.57;  W3: 347 ± 181.65; W5: 692 ± 373.97; W8: 716 ± 421.78; W10: 812 ± 475.80; W12: 692 ± 312.37 | No C/E  (BUT C/E found for MR). |
| **Raby et al. 2014** | RCT with flexible dose design | Total: 130  VEN: 64 (72%)  (43 patients discontinued) 🡪 21 (ITT)  *Cocaine-Dependency + Depression*  37 ± 8 | USA | From day 8 increased every week by 75 mg to reach 300 mg or the maximum tolerated dosage. | NA | NA | 477.82 ± 215.5 | No C/E found.  Excluded from meta-analysis because of differing indication. |
| **Sakolsky et al. 2011** |  | VEN: 119  *MDD* | USA | 37.5 mg for week 1, and for weeks 2 to 4, they received 75 mg, 112.5 mg, and 150 mg, respectively, with an optional increase from 150 to 225 mg at week 6. From 6 week through week 12, the average daily doses of study medications were 200.9 mg (SD = 35.2). | 123.0 ± 126.2,  CI 90% 59.0 - 85.6  **Resp.:** 108.6 ± 93.1 CI 90% 50.6-84.4  **Nonresp.:** 140.3 ± 156.2 CI90% 59.5-103.6 | 200.5 ± 125.7  CI90% 117.4-165.2  **Resp.:** 184.4 ± 119.8 CI90% 95.2-156.8 **Nonresp.:**  219.9 ± 130.8  CI90% 129.5-205.0 | 323.5 ± 195.1 CI90% 197.1- 272.7  **Resp.:** 293.0 ± 175.6 CI90% 162.0-258.1  **Nonresp.:**  360.2 ± 212.2  CI90% 215.3-337.4 | Positive C/E found;  Among those treated with VEN, higher exposure was associated with dizziness when standing up, cardiovascular, and dermatologic adverse effects. |
| **Tadic et al. 2016 inlcuding Engelmann et al. 2021** | RCT and post hoc analysis with flexible dose design | Total: 879  VEN: 97  (11 dropouts; 41 non-responders) (ITT)  EMC: 97 (43,3%)  TAU: 95 (45,3%)  *non-improvement of MDD after ESC treatment* EMC: 39.4 ± 11.5  TAU:  38.9 ± 12.2 | Germany | **EMC:**  Mean:  255.7 ± 62.9  **TAU:**  262.8 ± 62.6 | NA | NA | **EMC:** 460.8 ± 145.9 **TAU:** 460.7 ± 161.3 | No C/E investigated. |
| **Owens et al. 2008** | RCT with fixed dose design | Total: 86  *MDD* | US | 75-375 mg/day, forced titration upward at weekly and bi-weekly intervals to maximum. | NA | NA | NA | No C/E investigated. |
| **Aldosary et al.** | RCT with flexible dose design | VEN: 10 (70%)  *MDD*  41.3 ± 10.1 | Canada | 37.5 – 300 BID | NA | NA | 101 ± 69 | No C/E reported, study was not included in meta-analysis because of altered dose regiments. |

*S9. Study details concentration studies^3^.*

| **Authors, year** | **Study Design** | **Subjects**  **(% females) Indication**  **Mean Age** | **Country** | **Mean dose ± SD (range) (mg/d)** | **Mean VEN BL ± SD (ng/ml), if not specified other**  ***=converted from original** | **Mean ODV BL ± SD (ng/ml), if not specified other**  ***=converted from original** | **Mean AM BL ± SD (ng/ml), if not specified other**  ***=converted from original** | **Comment/Main outcome** |
| --- | --- | --- | --- | --- | --- | --- | --- | --- |
| **Cohort** | | | | | | | | |
| **Gex-Fabry et al. 2002 (D1)** | CS with fixed dose design | 35 (60%)  *moderate or severe depressive episode*  Median: 44 | Switzerland | 300 mg from day 3  BID | Median: 193 Range: 20-1294  Median +/- ratio: 1.14 | Median: 418 Range: 175-823  Median +/- ratio: 0.97 | Median: 677 Range: 202-1605 | Interindividual variability was larger for VEN (77%) than for ODV (33%) or AM (31%).  Gender differences: When compared with males, females displayed a significantly reduced ratio for the (–) enantiomer, but not the (+) enantiomer.  Study was not included in meta-analysis because of altered dose regimen. |
| **Grözinger et al. 2003** | CS with flexible dose design | retrospectively analyzed:  94 (73%)  VEN + MEL: 3 (66.67%)  MEL: 7  *NA* | Germany | 75-400 mg  150 mg (N=46)  225 mg (N=34) | NA | NA | NA | In this study a significant effect of melperone on the pharmacokinetics of VEN was found. |
| **Hefner et al. 2015** | CS with flexible dose design | 39 (54%)  *NA*  Median: 57 | Germany | NA | [ng/ml/mg] elevated CRP: 0.54 (0.11–3.07)  normal CRP: 0.59 (0.12–2.25) | [ng/ml/mg] elevated CRP: 1.06 (0.32–3.19)  normal CRP:0.95 (0.29–3.18) | [ng/ml/mg] elevated CRP: 1.76 (0.71–4.15)  normal CRP: 1.68 (0.59–3.94) | No significant differences were found between elevated CRP-group and normal CRP-group regarding PK. |
| **Silhan et al. 2018** | CS with flexible dose design | Total: 83 (77.1%)  VEN: 24 (NA%)  *Multiple diagnosis*  40.3 ± 12.2 | Czech Republic | 171.88 ± 81 | 124.9 ± 226.8 | 261.8 ± 174.3 | 386.7 ± 328.7 | TDM study to assess patients‘ adherence and conformity of antidepressant blood levels with published reference ranges. |
| **Tveit et al. 2020** | CS with flexible dose design | Total: 10 280  VEN: 2 326 (61%) individuals and 2978 (60%) samples  *NA* | Norway | >64 y Group  2007 (139 patients, 170 samples): geometric mean (95% CI): 150 (139-163)  2017 (289 (387)): GM(95%CI): 125 (118-133)  <65Group  2007 (1040(1341)): GM(95%CI): 176(171-181)  2017 (858(1080)): GM(95%CI): 157 (152-162) | NA | NA | >64 Group  2007 (119(148)): GM(95%CI): 340 (303-381)  2017 (261(344)): GM(95%CI): 310 (292-329)  <65 Group:  2007 (918(1163)): 243 (233-252)  2017 (708(874)): 259 (282-306) | This study investigates prescribing trends. |
| **Crosssectional Studies** | | | | | | | | |
| **Augustin et al. 2018** | CCS with flexible dose design | Total: 130;  VEN XR: 88  *NA*  57.49 ± 13.5 | Germany | 204.5 ± 62.7 | 124.3 ± 140.4  Median: 72.0  Median (arithmetic mean, SD)  0.35 (0.6, 0.6)  [(ng/mL)/(mg/day)] | 262.9 ± 119.3  Median: 260.5  Median (arithmetic mean, SD)  1.3 (1.4, 0.7) [(ng/mL)/(mg/day)] | 387.2 ± 168.8  Median: 342.2  Median (arithmetic mean, SD)  1.9 (2.0, 0.8) [(ng/mL)/(mg/day)] | No significant differences were found between the groups with regard to the PK of VEN (P = 0.123), ODV (P = 0.386), or AM (P = 0.731). Furthermore, C/Ds showed no differences in terms of C/D VEN (P =0.475), C/D ODV (P = 0.084), or C/D AM (P = 0.064). The MR was significantly lower in the AMLO group (P =0.029) compared with the control group. |
| **Chermá et al. 2008** | CCS dose design NA | Total: 76 (78%) VEN: 1  *multiple diagnosis*  84 | Sweden | 75 | NA | NA | 604 | Very small sample size. |
| **Fekete et al. 2020** | CCS dose design NA | Total: 953 (58%) <18 y: 26 (77%)  18-59 y: 637 (55%) ≥60 y: 290 (63%) Monotherapy: 37  *multiple diagnosis*  Range: 12-93  <18: Median 16, IQR: 2  18-59: Median 41, IQR: 23  >59: Median 71, IQR: 13 | Germany | Total range: 37,5-450 <18: Median 131,25 IQR 75; 18-59: Median 225, IQR 150; >59: Median 150, IQR 112,5 | <18: Median 42, IQR 70;  18-59: Median 71, IQR 111,8 ;  >59: Median 79, IQR 125 | 18: Median 122, IQR 98,75;  18-59: Med. 212, IQR 171,5;  >59: Median: 261, IQR 183,5 | <18: Median; IQR 176.0; 188.75;  18-59: M; IQR 312.0; 246.5;  >59: M.; IQR 369.5; 268.0 | Adult and elderly females showed higher CD/AM compared to males but not child/adolescents females. MPR stays the same over lifetime and compared beween genders - that supports that CYP2D6 activity may not change over the lifetime. |
| **Hansen et al. 2017** | CCS with flexible dose design | Total: 1 077 (69%)  *NA*  median: 45  IQR: 34-59  >64: 18% | Denmark | median: 225 (IQR 150-225) in detail: <150 mg (N=46) - median 75 ( IQR 75–75); 150–225 mg (N=807) - median 225 (IQR 150–225); >225 mg (N=224) - median 300 (IQR 300-300) | Median: 306 nmol/L (IQR 156-601)  *84,76 (43,21-166,48) | Median: 861 nmol/L (IQR 600-1260)  *226,58 (157,89-331,58) | NA | Females had a significantly higher dose-corrected serum concentration of parent compound, ODV and the sum of both compounds; In a post hoc analysis, there was verified that the observed association with age was consistent when stratifying by sex, and conversely, that the association with female sex was seen for both young and old patients. |
| **Hermann et al. 2008** | CCS with flexible dose design | 43 (47%)  *NA*  range: 21-83 median EMs: 39 median HEMs: 40 median PMs: 35 | Norway | Median EMs: 187.5 (range: 75-450) Median HEMs: 225 (75-300) Median PMs: 150 (75-375) | (nM/mg) Ems, HEMs, PMs  1.1 (0.3–5.2)  2.2 (0.6–10.0)  7.3 (3.1–20.0) | (nM/mg) Ems, HEMs, PMs  3.8 (1.8–6.1)  3.0 (1.1–6.4)  1.3 (0.2–2.3) | (nM/mg) Ems, HEMs, PMs  5.0 (2.3–8.6)  6.1 (2.6–13.5)  8.5 (4.2–20.1) | No significant difference in the active moiety (sum of venlafaxine and O-desmethylvenlafaxine) was observed between EM and HEM patients; However, the metabolic ratio (O-desmethylvenlafaxine/venlafaxine) was 50% lower in HEMs than in EMs (p<0.05), due to a twofold higher level of VEN in HEMs than in EMs (significant). The largest difference between genotype groups was observed for NDV. For this metabolite, serum concentration was 5.5-fold higher in HEMs (p<0.01) and 22-fold higher in PMs (p<0.001) than in EMs. |
| **Jukic et al. 2021** | CCS with flexible dose design | VEN: 1 742 (64%)  Median: 45  IQR 33-61 | Norway | Median: 150 (IQR 125-225) | NA | NA | NA | The CYP2D6 genotype significantly affected MRs for VEN. The difference in metabolic activity between CYP2D6 diplotypes is very pronounced and it is approximated by the MRs of risperidone, venlafaxine, and aripiprazole in a similar but not exactly the same fashion. |
| **Komahashi-Sasaki et al. 2020** | CCS with flexible dose design | Included VEN:  75 (63%)  No CYP2D6 Mutation: 35 (66%)  *multiple diagnosis*  Median: 49.0 Range: 20-84  No CYP2D6 Mutation:  Median 46  (range: 20-84) | Japan | Median: 75 Range: 37.5-225 | Median: 18.60 | Median: 59.10 | Median: 86.8 | Significant positive correlations were observed between the daily dose of VEN (corrected for body weight) and plasma concentrations of VEN and ODV. No significant relationship between ODV and VEN levels and number of CYP2D6 mutated alleles found. Significant difference between ODV/VEN ratios between different numbers of CP2D6 mutated alleles found. The steady-state plasma concentration of ODV correlated with sex - no plausible explanation for this correlation found. |
| **Kowalewski et al. 2019** | CCS with flexible dose design | VEN: 906 (59,9%) VEN+TRIM: 33  *multiple diagnosis*  46.01 ± 15.07 | Germany | Mean ± SD: 202.44 ± 79.37 | Median (range) 72.0 (1.6–1212.0) | Median (range) 162.40 (0.7–889.0) | Median (range) 267.5 (5.9–1370.0) | Plasma concentrations of active moiety (AM) were higher in the trimipramine group than in the control group. |
| **Kringen et al. 2020** | CCS with flexible dose design | 1000 (64%)  *NA*  Median: 46  (14-95) | Norway | Median: 150 Range: 75-225 | CYP2D6/CYP2C19:  C/D Mean (95%CI): NM/NM (N=367): 0.61 (0.44–0.78);  NM/IM (N=142) 0.78 (0.49–1.07);  IM/NM (N=237) 0.92 (0.65–1.19);  IM/IM (N=82):  1.17 (0.85–1.49) | 1.45 (1.29–1.61)  1.71 (1.44–1.99)  1.24 (0.99–1.50)  1.39 (1.08–1.69) | 2.09 (1.85–2.33)  2.53 (2.12–2.95)  2.18 (1.79–2.56)  2.57 (2.11–3.03) | A stepwise increase in both the dose-adjusted serum concentrations of venlafaxine and the sum of venlafaxine + ODV was observed in relation to decreased CYP2C19 metabolizer phenotypes. VEN was 12.9 fold increased in combined PMs and 1.9 fold increased in IM compared to combined NMs. In addition, ODV levels were more or less absent in combined PMs, whereas NDV was significantly increased in the same group, compared with combined NMs. |
| **Kuzin et al. 2018** | CCS with flexible dose design | non-PPI VEN: 906 Omeprazol: 40 Pantoprazol: 40  *NA* | Germany | 202 mg/day (SD) 79.3 | Median: 72.0 (range: 1.6–1212.0) | Median: 162.40 (range: 0.7–889.0) | Median: 267.5 (range: 5.9–1370.0) | BL of VEN, ODV and AM in the pantoprazole group were significantly higher than in the non-PPI group |
| **Mannheimer et al. 2016** | CCS with flexible dose design | RIS: 425  VEN: 498 (63%)  *NA*  Median: 46  (17-95) | Norway | Median: 15  (19-600) | NA | NA | NA | A RIS or VEN MR >1 strongly predicts CYP2D6 PMs. |
| **Mc Alpine et al. 2011** | CCS with flexible dose design | 95 (75,8%)  *NA*  43 (18–81) | US | NA | NA | NA | NA | Age was not significantly associated with the serum concentrations.  CYP2D6 and CYP2C19 scores are negatively associated with VEN BLs (after dose-adjustment in linear regression).  The CYP2D6 score was significantly positively associated with ODV blood concentration after adjusting for the dose of VEN, indicating that subjects carrying a higher number of CYP2D6 alleles producing active enzymes tended to have a higher concentration of ODV. CYP2C19 score was not found to be significantly associated with ODV concentrations. |
| **Paulzen et al. 2015** | CCS with flexible dose design | Total: 16 (62,5%)  *MDD*  60.88 ± 16.20 | Germany | 75-225 mg/day mean = 152.34 ± 67.73 mg/d | 20.00 to 530.00 ng/mL (mean = 171.25 ng/mL, SD = 145.64 ng/mL) | 19.00 to 503.00 ng/ mL (mean = 197.38 ng/mL, SD = 145.36 ng/mL) | mean 368.63 ng/mL (SD = 214.29 ng/mL; range, 98.00–675.00 ng/mL) | A significant correlation of the daily VEN dose and the BL VEN could be observed (r = 0.659, P = .005). ODV BLs were not significantly correlated to the daily dose of VEN. The VEN level in CSF showed a significant correlation to the daily dose of venlafaxine (r = 0.562, r2 = 0.316, P = .023). However, no correlation of CSF levels of ODV with the daily dose of VEN could be observed. |
| **Paulzen et al. 2018** | CCS with flexible dose design | VEN only: 905 (60%)  *Multiple diagnosis*  46.03 | Germany | Median: 225  (37.5-450) | Median: 72.0  (1.6-1212.0) | Median: 162.80 (0.7-889.0) | Median: 267.0 (5.9-1370.0) | An inhibiting effect for doxepine on VEN metabolism is suggested, while mirtazapine does not seem to have a considerable potential of interactions. |
| **Reis et al. 2009** | CCS with flexible dose design | VEN: 1 781 (60%)  *NA*  Median: 43  (15-94) | Norway | Median: 150 (9.4–675)  37.5mg - 18  75mg - 256  112.5mg - 42  150mg - 716  225mg - 391  300mg - 252  375mg - 34  450mg - 22 | Total:Median (10.-90. Percentile): *62.33 (12.74-249.31)  Median (10% 90%) 37.5mg: 35.18 (4,71 - 164,54)  Median (10% 90%) 75mg: 27.42 (6.37 - 139.34)  Median (10% 90%) 112.5mg: 167 (49-1011)  Median (10% 90%) 150 mg: 179 (46-677)  Median (10% 90%) 225mg: 327 (83-1017)  Median (10% 90%) 300mg: 418 (104-1391)  Median (10% 90%)375mg: 669 (118-1760)  Median (10%90%) 450mg: 334 (103-1419) | Median (10% 90%)37,5 mg: 256 (48-718)  Median (10% 90%)75mg: 373 (160-822)  Median (10% 90%)112,5mg: 509 (131-1069)  Median (10% 90%)150mg: 535 (221-1085)  Median (10% 90%)225mg: 711 (313-1480)  Median (10% 90%)300mg: 858 (381-1645)  Median (10% 90%) 375mg: 1078 (407-1993)  Median (10% 90%) 450mg: 766 (447-1160) | Median (10% 90%) 37,5mg: 345 (188-1024)  Median (10% 90%)75mg: 487 (245-1200)  Median (10% 90%) 112,5mg: 827 (292-1489)  Median (10% 90%) 150mg: 787 (382-1590)  Median (10% 90%)225mg: 1193 (494-2277)  Median (10% 90%) 300mg: 1407 (674-2635)  Median (10% 90%) 375mg: 1561 (862-3605)  Median (10% 90%) 450mg: 1292 (712-2689) | Significant differences in median parent substance concentrations between men and women, and between old and young patients.  The MPR for VEN was significantly higher in men than in women. |
| **Reis et al. 2002** | CCS with flexible dose design | Total: 187 (68%)  VEN: 17  *NA*  Median: 51.7  (4 age groups) | Sweden | Median: 150 mg/d (37.5 mg/d to 412.5 mg/d) IQ: 112.5-225 | 37.5mg (n=6) 50 ± 40 (7-101)  75mg (34) 270 ± 335 (17-1540)  112.5(8) 388 ± 498 (14-1400)  150 (68) 393 ± 836 (96-477)  225 (29)643 ± 1021 (34-5121)  300 (23) 562 ± 394 (74-1580)  Median (range):  37.5mg (n=6) 42 (11-99)  75mg (34) 111 (47-480)  112.5(8) 169 (59-620)  150 (68) 225 (96-477)  225 (29)362 (171-563) | 37.5mg (n=6) 392 ± 237 (222-811)  75mg (34) 441±246 (62-987)  112.5(8) 541 ± 293 (270-1002)  150 (68) 705 ± 358 (62-1560)  225 (29) 881 ± 503 (240-2796)  Median (range):  37.5mg (n=6) 267 (233-676)  75mg (34) 377 (243-651)  112.5(8)418 (334-776)  150 (68) 618 (434-951)  225 (29) 880 (592-989) | 37,5mg: 442 ± 169,95*  75mg: 711 ± 293,89 *  112,5mg: 929 ± 408,57*  150mg: 1903 ± 643,06*  225mg: 1524 ± 804,81*  Median (range):  NA | **No relationship was seen between dose and VEN.**  None of the ratios in PE (ODV/VEN, NDV/VEN, or DDV/VEN) were Gaussian-distributed.  A clear age-related difference was found in which the older patients had higher C/D VEN and C/D ODV values than the younger ones.  No differences in C/D VEN values or in any of the metabolite/VEN ratios were found between smokers and non-smokers.  Comparing the four therapy groups, no difference between the metabolite concentrations could be found. The median ODV/VEN ratio was lower for “Polypharmacy” than for “Polycombination CNS”. In the second group, in which VEN was combined with a somatic drug, the two most prominent deviations from “Monotherapy” were the combinations with alimemazine and omeprazole. In addition to omeprazole, all of them medicated with at least two CNS drugs suspected of interacting with VEN; i.e., being metabolized by CYP2D6. |
| **Schoretsanitis et al. 2018** | CCS with flexible dose design | VEN ER: 737 (61,1%)  *Multiple diagnosis*  45.44 ± 14.62 | Germany | 205.42 ±81.07 (range: 37.5–450.0)  Median: 225.00  BMI<20: 191.19 ±77.37  BMI 20-29: 202.2 ±80.31  BMI>30: 215.93 ± 82.79 | Median (BMI<20): 88 (range 3-699)  Median (BMI 20-29): 66 (1.6-725)  Median (BMI>30): 74 (2.7-1212.0)  C/D:  BMIlow: 0.5  BMInormal: 0.35  BMIhigh: 0.4 | Median (BMI<20): 190 (range: 2.5-683)  Median (BMI 20-29): 167.5 (range: 0.8-613)  Median (BMI>30): 149 (0.7-889)  C/D:  BMIlow: 1.06  BMImed: 0.87  BMIhigh: 0.79 | Median (BMI<20): 300 (range: 46-1034)  Median (BMI 20-29): 267.5 (7,7-1115)  Median (BMI>30): 261,5 (5,9-1370,0)  C/D: BMIlow: 1.63  BMImed: 1.37  BMIhigh: 1.34 | A weak but significant negative correlation was detected between body weight and AM as well as between body weight and ODV concentration values; weak but significant negative correlations were detected between body weight and C/D values of all pharmacokinetic parameters.  BMI was correlated negatively with the C/D AM and C/D ODV levels. |
| **Scherf-Clavel et al. 2019** | CCS with flexible dose design | VEN: 534 (59%)  *"any psychiatric diagnosis"*  47.36 ± 16.48 | Germany | 194.03 ± 83.47 (range: 37.5-450) | 136.48±134.55  C/D:  0.75 ± 0.74 | 211.18±139.00  C/D:  1.16± 0.65 | 347.65±219.27  C/D:  1.90± 1.06 | Higher C/D of VEN, ODV and sum values in older patients and women  The ratio ODV/VEN was significantly higher in men compared with women  Smoking did not affect anything. |
| **Sigurdsson et al. 2014** | CCS with flexible dose design | 1 417 (61%)  *Multiple diagnosis*  49.4 ± 15.4 | Germany | 207.8 ± 78.8 (37.5–450.0) | 140.2 ± 147.7 (1.0–1459.0) | 212.7 ± 133.6 (0.60–1052.1) | 352.9 ± 212.2 (22.0–1557.0)  C/D: 1.79 ± 1.09 (0.10–11.97) (ng/mL/mg) | Older patients tend to have significantly C/Ds than patients aged 18 to 40.  Females received a significantly lower dosage of VEN compared with males, but females demonstrated a significantly higher C/D AM.  Old aged female patients had by mean 70% higher dose-adjusted drug concentrations than young male patients.  In regard to venlafaxine metabolites, BMI only correlated significantly with ODV concentration among younger female patients. |
| **Unterecker et al. 2011** | CCS dose design NA | VEN: 24 (83%)  *NA*  50.6 ± 20.6 | Germany | 200.0 ± 105.3 (75–375) | NA | NA | 418.4 ± 251.3 (82–1034) |  |
| **Unterecker et al. 2012** | CCS with flexible dose design | 478 (64%)  *NA*  49.1 ± 15.5 | Germany | 199.1 ± 87.8 mg; range 37.5–525 mg, median 150 mg | NA | NA | 387.0 , SD 236.8, range: 7 - 1 656 | Applied dose and serum level of VEN and ODV correlated only moderately. Stronger correlation was found for the sum of VEN and ODV.  The C/D AM in female non-smokers older than 60 years (n = 34, mean value 3.06 ng/mL/mg, SD 1.88) was nearly twice as high as that in male smokers up to 60 years (n = 27, mean value 1.65 ng/mL/mg, SD 0.94). |
| **Unterecker et al. 2014** | CCS with flexible dose design | Total: 178  Brand VEN:  110 (53%)  Generic VEN:  68 (59%)  after matching:  2 groups of 35 patients (63%)  *NA*  Brand: 47.3 ± 17.0  Gen.: 38.4 ± 14.5  after matching: 37.8 ± 13.1 vs  37.8 ± 13.2 | Germany | Brand: 240.5 mg (SD 96.0, range 75–600)  Generic: 209.2 mg (SD 85.1, range 37.5–375)  after matching:  212.1 ± 83.8 (37.5–375) vs  226.1 ± 104.7 (75–450) mg | after matching  Gen: 95.6 ± 52.0 (10–235)  Brand: 93.6 ± 61.7 (13–236) | Gen: 202.2 ± 104.2 (16–476)  Brand: 190.5 ± 98.0 (43–524) | Generic:  320.4 ± 161.0  (26–717)  Brand:  375.5 ± 214.6,  (56–1443)  after matching: Gen: 297.8 ± 148.8 (26–711)  Brand: 284.1 ± 152.1 (56–760) | No differences between generic and brand groups were found. |
| **Unterecker et al. 2014 (Interaction of VA with Dox and Ven)** | CCS with flexible dose design | VEN+VA: 41  VEN: 41  VEN+VA: 45.9±16.2  VEN: 45.7±15.8  *NA* | Germany | VEN+VA: 209.1±81.5 (75–375)  VEN: 206.7±80.0 (75–375) | VEN+VA: 131.1±110.5 (24–488)  VEN: 117.4±76.2 (24–409) | VEN+VA: 271.7±139.6 (52–720)  VEN: 211.1±93.1 (57–516) | VEN+VA: 402.8±210.2 (76–981)  VEN: 328.5±130.4 (115–700) | In patients receiving VEN and valproic acid, the mean serum concentration of ODV was higher than that in patients treated with VEN alone. For C/D of AM, there only was a trend for a difference. |
| **Waade et al. 2014** | CCS with flexible dose design | VEN: 462 samples from 255 patients  *NA* | Norway | range: 18.75–900 | C/D:  Data given in this group order: <40, 40-65, >65 y  2.4 (1.6–3.7) 6.4 (4.7–8.7) 18.8 (9.4–37.6) (PM)  1.7 (1.2–2.4) 2.0 (1.5–2.6) 2.7 (1.8–4.0) (HEM)  1.2 (1.0–1.5) 1.0 (0.8–1.3) 1.4 (0.9–2.1) (EM) | C/D:  Data given in this group order: <40, 40-65, >65 y  1.3 (0.8–2.2) 1.1 (0.7–1.6) 0.6 (0.2–1.3) (PM)  3.2 (2.7–3.9) 3.3 (2.8–3.8) 4.8 (3.8–5.9) (HEM)  3.4 (3.0–3.8) 3.5 (3.1–4.0) 6.1 (4.8–7.8) (EM) | C/D:  Data given in this group order: <40, 40-65, >65 y  3.9 (2.8–5.4) 8.0 (6.2–10.2) 19.7 (11.4–34.2) (PM)  5.3 (4.3–6.5) 5.7 (4.8–6.8) 8.2 (6.5–10.4) (HEM)  5.1 (4.5–5.7) 5.0 (4.4–5.7) 7.9 (6.3–9.9) (EM) | In CYP2D6 PMs, the mean C/D ratio of VEN was about 8-fold and 2.5-fold higher in patients >65 and 40– 65 years, respectively, compared with those <40 years. In comparison, the respective differences in mean C/D ratios of VEN were much less pronounced and not significantly different between CYP2D6 HEMs and EMs . Similar effect of age was observed for VEN+ODV C/D. |
| **van der Weide et al. 2005** | CCS with flexible dose design | VEN: 210 samples of 92 patients  *NA* | Netherlands | NA | NA | NA | NA | There is a significant correlation between the CYP2D6 genotype and the log(VEN/ODV). Gender and age were not significantly different among the 4 genotype groups (P = 0.657 and 0.962 respectively). |
| **Wang et al. 2020** | CCS with flexible dose design | 91 samples from 80  individuals (62,5%)  *multiple diagnosis*  40.1 ± 17.7 | China | 171.4 ± 59.5 | 163.3 ± 136.7  Median:  130.4 (range: 4.9, 888)  C/D:  1.0 ± 0.8  Median (range): 0.8 (0.1, 4.8) | 181.1 ± 93.1  Median:  170.5 (range: 19.4, 527.4)  C/D:  1.1 ± 0.6  Median (range):  1 (0.2, 4) | 341.1 ± 176.3  Median:  317.0 (range: 28.6, 945.0)  C/D:  2.1 ± 1.0  Median (range):  2 (0.5, 5.9) | Remarkable sex differences were found in body weights and BLs of VEN + ODV. Females had an average 13% smaller body weight and an average 43% larger serum concentration of VEN + ODV than males.  It was detected a negative correlation between VEN dosage and the C/D of ODV, and between VEN concentrations and MR, indicating a possible partial saturation of the metabolic capacity. |
| **Warrings et al. 2021** | CCS with flexible dose design | VEN: 380 (58.4%)  *NA*  48.1 ± 16.1 | Germany | 196.29 ± 84.2 (37.5–450) | 130,6 ± 125.1 (5–966)  C/D:  0.70 ± 0.61 (0.04–4.05) | 217.0 ± 135.8 (10–1079)  C/D:  1.18 ± 0.67 (0.09–4.57) | 347.6 ± 212.6 (22–1712)  C/D:  1.88 ± 0.99 (0.15–6.39) | C/D ODV and C/D AM of VEN were negatively associated with BMI. |
| **Ganesh et l. 2021** | CCS  dose design NA | VEN: 193 (65%)  *NA*  Median: 48 | The Netherlands | Median: 131 (IQR: 75 – 150) | NA | NA | NA | A strong correlation between log([VENL]/[DVEN]) ≥ 0.1 and CYP2D6 PM phenotype prediction was found. |
| **RCTs** | | | | | | | | |
| **Boulton et al. 2010**  **Berman et al. 2007 + Marcus et al. 2008 (study 3 + 4 )** | RCT and post hoc analysis with flexible dose design | Total: 498  *Berman et al.*  **Responders:** 262  PLC + VEN: 176  (19 dropouts)  *Marcus et al.*  PLC + VEN: 190  *MDD*  *Berman et al.*  44.2 ± 109  *Marcus et al.*  44.4 ± 10.7 | US | VEN ER (150 - 225 mg/day) | NA | NA | NA | The pooled pharmacokinetic analysis from the two safety and efficacy studies in patients with MDD (Studies 3 and 4) did not demonstrate any meaningful pharmacokinetic effects of VEN on the five ADRs investigated. |

*S10. Study details neuroimaging studies^3^.*

| **Authors, year** | **Study Design** | **Subjects**  **(% females) Indication**  **Mean Age** | **Country** | **Mean dose ± SD (range) (mg/d)** | **Mean VEN BL ± SD (ng/ml), if not specified other**  ***=converted from original** | **Mean ODV BL ± SD (ng/ml), if not specified other**  ***=converted from original** | **Mean AM BL ± SD (ng/ml), if not specified other**  ***=converted from original** | **Comment/Main outcome** |
| --- | --- | --- | --- | --- | --- | --- | --- | --- |
| **Cohort** | | | | | | | | |
| **Gex-Fabry et al. 2002 (D1)** | CS with fixed dose design | 35 (60%)  *moderate or severe depressive episode*  Median: 44 | Switzerland | 300 mg from day 3  BID | Median: 193 Range: 20-1294  Median +/- ratio: 1.14 | Median: 418 Range: 175-823  Median +/- ratio: 0.97 | Median: 677 Range: 202-1605 | Interindividual variability was larger for VEN (77%) than for ODV (33%) or AM (31%).  Gender differences: When compared with males, females displayed a significantly reduced ratio for the (–) enantiomer, but not the (+) enantiomer.  Study was not included in meta-analysis because of altered dose regimen. |
| **Grözinger et al. 2003** | CS with flexible dose design | retrospectively analyzed:  94 (73%)  VEN + MEL: 3 (66.67%)  MEL: 7  *NA* | Germany | 75-400 mg  150 mg (N=46)  225 mg (N=34) | NA | NA | NA | In this study a significant effect of melperone on the pharmacokinetics of VEN was found. |
| **Hefner et al. 2015** | CS with flexible dose design | 39 (54%)  *NA*  Median: 57 | Germany | NA | [ng/ml/mg] elevated CRP: 0.54 (0.11–3.07)  normal CRP: 0.59 (0.12–2.25) | [ng/ml/mg] elevated CRP: 1.06 (0.32–3.19)  normal CRP:0.95 (0.29–3.18) | [ng/ml/mg] elevated CRP: 1.76 (0.71–4.15)  normal CRP: 1.68 (0.59–3.94) | No significant differences were found between elevated CRP-group and normal CRP-group regarding PK. |
| **Silhan et al. 2018** | CS with flexible dose design | Total: 83 (77.1%)  VEN: 24 (NA%)  *Multiple diagnosis*  40.3 ± 12.2 | Czech Republic | 171.88 ± 81 | 124.9 ± 226.8 | 261.8 ± 174.3 | 386.7 ± 328.7 | TDM study to assess patients‘ adherence and conformity of antidepressant blood levels with published reference ranges. |
| **Tveit et al. 2020** | CS with flexible dose design | Total: 10 280  VEN: 2 326 (61%) individuals and 2978 (60%) samples  *NA* | Norway | >64 y Group  2007 (139 patients, 170 samples): geometric mean (95% CI): 150 (139-163)  2017 (289 (387)): GM(95%CI): 125 (118-133)  <65Group  2007 (1040(1341)): GM(95%CI): 176(171-181)  2017 (858(1080)): GM(95%CI): 157 (152-162) | NA | NA | >64 Group  2007 (119(148)): GM(95%CI): 340 (303-381)  2017 (261(344)): GM(95%CI): 310 (292-329)  <65 Group:  2007 (918(1163)): 243 (233-252)  2017 (708(874)): 259 (282-306) | This study investigates prescribing trends. |
| **Crosssectional Studies** | | | | | | | | |
| **Augustin et al. 2018** | CCS with flexible dose design | Total: 130;  VEN XR: 88  *NA*  57.49 ± 13.5 | Germany | 204.5 ± 62.7 | 124.3 ± 140.4  Median: 72.0  Median (arithmetic mean, SD)  0.35 (0.6, 0.6)  [(ng/mL)/(mg/day)] | 262.9 ± 119.3  Median: 260.5  Median (arithmetic mean, SD)  1.3 (1.4, 0.7) [(ng/mL)/(mg/day)] | 387.2 ± 168.8  Median: 342.2  Median (arithmetic mean, SD)  1.9 (2.0, 0.8) [(ng/mL)/(mg/day)] | No significant differences were found between the groups with regard to the PK of VEN (P = 0.123), ODV (P = 0.386), or AM (P = 0.731). Furthermore, C/Ds showed no differences in terms of C/D VEN (P =0.475), C/D ODV (P = 0.084), or C/D AM (P = 0.064). The MR was significantly lower in the AMLO group (P =0.029) compared with the control group. |
| **Chermá et al. 2008** | CCS dose design NA | Total: 76 (78%) VEN: 1  *multiple diagnosis*  84 | Sweden | 75 | NA | NA | 604 | Very small sample size. |
| **Fekete et al. 2020** | CCS dose design NA | Total: 953 (58%) <18 y: 26 (77%)  18-59 y: 637 (55%) ≥60 y: 290 (63%) Monotherapy: 37  *multiple diagnosis*  Range: 12-93  <18: Median 16, IQR: 2  18-59: Median 41, IQR: 23  >59: Median 71, IQR: 13 | Germany | Total range: 37,5-450 <18: Median 131,25 IQR 75; 18-59: Median 225, IQR 150; >59: Median 150, IQR 112,5 | <18: Median 42, IQR 70;  18-59: Median 71, IQR 111,8 ;  >59: Median 79, IQR 125 | 18: Median 122, IQR 98,75;  18-59: Med. 212, IQR 171,5;  >59: Median: 261, IQR 183,5 | <18: Median; IQR 176.0; 188.75;  18-59: M; IQR 312.0; 246.5;  >59: M.; IQR 369.5; 268.0 | Adult and elderly females showed higher CD/AM compared to males but not child/adolescents females. MPR stays the same over lifetime and compared beween genders - that supports that CYP2D6 activity may not change over the lifetime. |
| **Hansen et al. 2017** | CCS with flexible dose design | Total: 1 077 (69%)  *NA*  median: 45  IQR: 34-59  >64: 18% | Denmark | median: 225 (IQR 150-225) in detail: <150 mg (N=46) - median 75 ( IQR 75–75); 150–225 mg (N=807) - median 225 (IQR 150–225); >225 mg (N=224) - median 300 (IQR 300-300) | Median: 306 nmol/L (IQR 156-601)  *84,76 (43,21-166,48) | Median: 861 nmol/L (IQR 600-1260)  *226,58 (157,89-331,58) | NA | Females had a significantly higher dose-corrected serum concentration of parent compound, ODV and the sum of both compounds; In a post hoc analysis, there was verified that the observed association with age was consistent when stratifying by sex, and conversely, that the association with female sex was seen for both young and old patients. |
| **Hermann et al. 2008** | CCS with flexible dose design | 43 (47%)  *NA*  range: 21-83 median EMs: 39 median HEMs: 40 median PMs: 35 | Norway | Median EMs: 187.5 (range: 75-450) Median HEMs: 225 (75-300) Median PMs: 150 (75-375) | (nM/mg) Ems, HEMs, PMs  1.1 (0.3–5.2)  2.2 (0.6–10.0)  7.3 (3.1–20.0) | (nM/mg) Ems, HEMs, PMs  3.8 (1.8–6.1)  3.0 (1.1–6.4)  1.3 (0.2–2.3) | (nM/mg) Ems, HEMs, PMs  5.0 (2.3–8.6)  6.1 (2.6–13.5)  8.5 (4.2–20.1) | No significant difference in the active moiety (sum of venlafaxine and O-desmethylvenlafaxine) was observed between EM and HEM patients; However, the metabolic ratio (O-desmethylvenlafaxine/venlafaxine) was 50% lower in HEMs than in EMs (p<0.05), due to a twofold higher level of VEN in HEMs than in EMs (significant). The largest difference between genotype groups was observed for NDV. For this metabolite, serum concentration was 5.5-fold higher in HEMs (p<0.01) and 22-fold higher in PMs (p<0.001) than in EMs. |
| **Jukic et al. 2021** | CCS with flexible dose design | VEN: 1 742 (64%)  Median: 45  IQR 33-61 | Norway | Median: 150 (IQR 125-225) | NA | NA | NA | The CYP2D6 genotype significantly affected MRs for VEN. The difference in metabolic activity between CYP2D6 diplotypes is very pronounced and it is approximated by the MRs of risperidone, venlafaxine, and aripiprazole in a similar but not exactly the same fashion. |
| **Komahashi-Sasaki et al. 2020** | CCS with flexible dose design | Included VEN:  75 (63%)  No CYP2D6 Mutation: 35 (66%)  *multiple diagnosis*  Median: 49.0 Range: 20-84  No CYP2D6 Mutation:  Median 46  (range: 20-84) | Japan | Median: 75 Range: 37.5-225 | Median: 18.60 | Median: 59.10 | Median: 86.8 | Significant positive correlations were observed between the daily dose of VEN (corrected for body weight) and plasma concentrations of VEN and ODV. No significant relationship between ODV and VEN levels and number of CYP2D6 mutated alleles found. Significant difference between ODV/VEN ratios between different numbers of CP2D6 mutated alleles found. The steady-state plasma concentration of ODV correlated with sex - no plausible explanation for this correlation found. |
| **Kowalewski et al. 2019** | CCS with flexible dose design | VEN: 906 (59,9%) VEN+TRIM: 33  *multiple diagnosis*  46.01 ± 15.07 | Germany | Mean ± SD: 202.44 ± 79.37 | Median (range) 72.0 (1.6–1212.0) | Median (range) 162.40 (0.7–889.0) | Median (range) 267.5 (5.9–1370.0) | Plasma concentrations of active moiety (AM) were higher in the trimipramine group than in the control group. |
| **Kringen et al. 2020** | CCS with flexible dose design | 1000 (64%)  *NA*  Median: 46  (14-95) | Norway | Median: 150 Range: 75-225 | CYP2D6/CYP2C19:  C/D Mean (95%CI): NM/NM (N=367): 0.61 (0.44–0.78);  NM/IM (N=142) 0.78 (0.49–1.07);  IM/NM (N=237) 0.92 (0.65–1.19);  IM/IM (N=82):  1.17 (0.85–1.49) | 1.45 (1.29–1.61)  1.71 (1.44–1.99)  1.24 (0.99–1.50)  1.39 (1.08–1.69) | 2.09 (1.85–2.33)  2.53 (2.12–2.95)  2.18 (1.79–2.56)  2.57 (2.11–3.03) | A stepwise increase in both the dose-adjusted serum concentrations of venlafaxine and the sum of venlafaxine + ODV was observed in relation to decreased CYP2C19 metabolizer phenotypes. VEN was 12.9 fold increased in combined PMs and 1.9 fold increased in IM compared to combined NMs. In addition, ODV levels were more or less absent in combined PMs, whereas NDV was significantly increased in the same group, compared with combined NMs. |
| **Kuzin et al. 2018** | CCS with flexible dose design | non-PPI VEN: 906 Omeprazol: 40 Pantoprazol: 40  *NA* | Germany | 202 mg/day (SD) 79.3 | Median: 72.0 (range: 1.6–1212.0) | Median: 162.40 (range: 0.7–889.0) | Median: 267.5 (range: 5.9–1370.0) | BL of VEN, ODV and AM in the pantoprazole group were significantly higher than in the non-PPI group |
| **Mannheimer et al. 2016** | CCS with flexible dose design | RIS: 425  VEN: 498 (63%)  *NA*  Median: 46  (17-95) | Norway | Median: 15  (19-600) | NA | NA | NA | A RIS or VEN MR >1 strongly predicts CYP2D6 PMs. |
| **Mc Alpine et al. 2011** | CCS with flexible dose design | 95 (75,8%)  *NA*  43 (18–81) | US | NA | NA | NA | NA | Age was not significantly associated with the serum concentrations.  CYP2D6 and CYP2C19 scores are negatively associated with VEN BLs (after dose-adjustment in linear regression).  The CYP2D6 score was significantly positively associated with ODV blood concentration after adjusting for the dose of VEN, indicating that subjects carrying a higher number of CYP2D6 alleles producing active enzymes tended to have a higher concentration of ODV. CYP2C19 score was not found to be significantly associated with ODV concentrations. |
| **Paulzen et al. 2015** | CCS with flexible dose design | Total: 16 (62,5%)  *MDD*  60.88 ± 16.20 | Germany | 75-225 mg/day mean = 152.34 ± 67.73 mg/d | 20.00 to 530.00 ng/mL (mean = 171.25 ng/mL, SD = 145.64 ng/mL) | 19.00 to 503.00 ng/ mL (mean = 197.38 ng/mL, SD = 145.36 ng/mL) | mean 368.63 ng/mL (SD = 214.29 ng/mL; range, 98.00–675.00 ng/mL) | A significant correlation of the daily VEN dose and the BL VEN could be observed (r = 0.659, P = .005). ODV BLs were not significantly correlated to the daily dose of VEN. The VEN level in CSF showed a significant correlation to the daily dose of venlafaxine (r = 0.562, r2 = 0.316, P = .023). However, no correlation of CSF levels of ODV with the daily dose of VEN could be observed. |
| **Paulzen et al. 2018** | CCS with flexible dose design | VEN only: 905 (60%)  *Multiple diagnosis*  46.03 | Germany | Median: 225  (37.5-450) | Median: 72.0  (1.6-1212.0) | Median: 162.80 (0.7-889.0) | Median: 267.0 (5.9-1370.0) | An inhibiting effect for doxepine on VEN metabolism is suggested, while mirtazapine does not seem to have a considerable potential of interactions. |
| **Reis et al. 2009** | CCS with flexible dose design | VEN: 1 781 (60%)  *NA*  Median: 43  (15-94) | Norway | Median: 150 (9.4–675)  37.5mg - 18  75mg - 256  112.5mg - 42  150mg - 716  225mg - 391  300mg - 252  375mg - 34  450mg - 22 | Total:Median (10.-90. Percentile): *62.33 (12.74-249.31)  Median (10% 90%) 37.5mg: 35.18 (4,71 - 164,54)  Median (10% 90%) 75mg: 27.42 (6.37 - 139.34)  Median (10% 90%) 112.5mg: 167 (49-1011)  Median (10% 90%) 150 mg: 179 (46-677)  Median (10% 90%) 225mg: 327 (83-1017)  Median (10% 90%) 300mg: 418 (104-1391)  Median (10% 90%)375mg: 669 (118-1760)  Median (10%90%) 450mg: 334 (103-1419) | Median (10% 90%)37,5 mg: 256 (48-718)  Median (10% 90%)75mg: 373 (160-822)  Median (10% 90%)112,5mg: 509 (131-1069)  Median (10% 90%)150mg: 535 (221-1085)  Median (10% 90%)225mg: 711 (313-1480)  Median (10% 90%)300mg: 858 (381-1645)  Median (10% 90%) 375mg: 1078 (407-1993)  Median (10% 90%) 450mg: 766 (447-1160) | Median (10% 90%) 37,5mg: 345 (188-1024)  Median (10% 90%)75mg: 487 (245-1200)  Median (10% 90%) 112,5mg: 827 (292-1489)  Median (10% 90%) 150mg: 787 (382-1590)  Median (10% 90%)225mg: 1193 (494-2277)  Median (10% 90%) 300mg: 1407 (674-2635)  Median (10% 90%) 375mg: 1561 (862-3605)  Median (10% 90%) 450mg: 1292 (712-2689) | Significant differences in median parent substance concentrations between men and women, and between old and young patients.  The MPR for VEN was significantly higher in men than in women. |
| **Reis et al. 2002** | CCS with flexible dose design | Total: 187 (68%)  VEN: 17  *NA*  Median: 51.7  (4 age groups) | Sweden | Median: 150 mg/d (37.5 mg/d to 412.5 mg/d) IQ: 112.5-225 | 37.5mg (n=6) 50 ± 40 (7-101)  75mg (34) 270 ± 335 (17-1540)  112.5(8) 388 ± 498 (14-1400)  150 (68) 393 ± 836 (96-477)  225 (29)643 ± 1021 (34-5121)  300 (23) 562 ± 394 (74-1580)  Median (range):  37.5mg (n=6) 42 (11-99)  75mg (34) 111 (47-480)  112.5(8) 169 (59-620)  150 (68) 225 (96-477)  225 (29)362 (171-563) | 37.5mg (n=6) 392 ± 237 (222-811)  75mg (34) 441±246 (62-987)  112.5(8) 541 ± 293 (270-1002)  150 (68) 705 ± 358 (62-1560)  225 (29) 881 ± 503 (240-2796)  Median (range):  37.5mg (n=6) 267 (233-676)  75mg (34) 377 (243-651)  112.5(8)418 (334-776)  150 (68) 618 (434-951)  225 (29) 880 (592-989) | 37,5mg: 442 ± 169,95*  75mg: 711 ± 293,89 *  112,5mg: 929 ± 408,57*  150mg: 1903 ± 643,06*  225mg: 1524 ± 804,81*  Median (range):  NA | **No relationship was seen between dose and VEN.**  None of the ratios in PE (ODV/VEN, NDV/VEN, or DDV/VEN) were Gaussian-distributed.  A clear age-related difference was found in which the older patients had higher C/D VEN and C/D ODV values than the younger ones.  No differences in C/D VEN values or in any of the metabolite/VEN ratios were found between smokers and non-smokers.  Comparing the four therapy groups, no difference between the metabolite concentrations could be found. The median ODV/VEN ratio was lower for “Polypharmacy” than for “Polycombination CNS”. In the second group, in which VEN was combined with a somatic drug, the two most prominent deviations from “Monotherapy” were the combinations with alimemazine and omeprazole. In addition to omeprazole, all of them medicated with at least two CNS drugs suspected of interacting with VEN; i.e., being metabolized by CYP2D6. |
| **Schoretsanitis et al. 2018** | CCS with flexible dose design | VEN ER: 737 (61,1%)  *Multiple diagnosis*  45.44 ± 14.62 | Germany | 205.42 ±81.07 (range: 37.5–450.0)  Median: 225.00  BMI<20: 191.19 ±77.37  BMI 20-29: 202.2 ±80.31  BMI>30: 215.93 ± 82.79 | Median (BMI<20): 88 (range 3-699)  Median (BMI 20-29): 66 (1.6-725)  Median (BMI>30): 74 (2.7-1212.0)  C/D:  BMIlow: 0.5  BMInormal: 0.35  BMIhigh: 0.4 | Median (BMI<20): 190 (range: 2.5-683)  Median (BMI 20-29): 167.5 (range: 0.8-613)  Median (BMI>30): 149 (0.7-889)  C/D:  BMIlow: 1.06  BMImed: 0.87  BMIhigh: 0.79 | Median (BMI<20): 300 (range: 46-1034)  Median (BMI 20-29): 267.5 (7,7-1115)  Median (BMI>30): 261,5 (5,9-1370,0)  C/D: BMIlow: 1.63  BMImed: 1.37  BMIhigh: 1.34 | A weak but significant negative correlation was detected between body weight and AM as well as between body weight and ODV concentration values; weak but significant negative correlations were detected between body weight and C/D values of all pharmacokinetic parameters.  BMI was correlated negatively with the C/D AM and C/D ODV levels. |
| **Scherf-Clavel et al. 2019** | CCS with flexible dose design | VEN: 534 (59%)  *"any psychiatric diagnosis"*  47.36 ± 16.48 | Germany | 194.03 ± 83.47 (range: 37.5-450) | 136.48±134.55  C/D:  0.75 ± 0.74 | 211.18±139.00  C/D:  1.16± 0.65 | 347.65±219.27  C/D:  1.90± 1.06 | Higher C/D of VEN, ODV and sum values in older patients and women  The ratio ODV/VEN was significantly higher in men compared with women  Smoking did not affect anything. |
| **Sigurdsson et al. 2014** | CCS with flexible dose design | 1 417 (61%)  *Multiple diagnosis*  49.4 ± 15.4 | Germany | 207.8 ± 78.8 (37.5–450.0) | 140.2 ± 147.7 (1.0–1459.0) | 212.7 ± 133.6 (0.60–1052.1) | 352.9 ± 212.2 (22.0–1557.0)  C/D: 1.79 ± 1.09 (0.10–11.97) (ng/mL/mg) | Older patients tend to have significantly C/Ds than patients aged 18 to 40.  Females received a significantly lower dosage of VEN compared with males, but females demonstrated a significantly higher C/D AM.  Old aged female patients had by mean 70% higher dose-adjusted drug concentrations than young male patients.  In regard to venlafaxine metabolites, BMI only correlated significantly with ODV concentration among younger female patients. |
| **Unterecker et al. 2011** | CCS dose design NA | VEN: 24 (83%)  *NA*  50.6 ± 20.6 | Germany | 200.0 ± 105.3 (75–375) | NA | NA | 418.4 ± 251.3 (82–1034) |  |
| **Unterecker et al. 2012** | CCS with flexible dose design | 478 (64%)  *NA*  49.1 ± 15.5 | Germany | 199.1 ± 87.8 mg; range 37.5–525 mg, median 150 mg | NA | NA | 387.0 , SD 236.8, range: 7 - 1 656 | Applied dose and serum level of VEN and ODV correlated only moderately. Stronger correlation was found for the sum of VEN and ODV.  The C/D AM in female non-smokers older than 60 years (n = 34, mean value 3.06 ng/mL/mg, SD 1.88) was nearly twice as high as that in male smokers up to 60 years (n = 27, mean value 1.65 ng/mL/mg, SD 0.94). |
| **Unterecker et al. 2014** | CCS with flexible dose design | Total: 178  Brand VEN:  110 (53%)  Generic VEN:  68 (59%)  after matching:  2 groups of 35 patients (63%)  *NA*  Brand: 47.3 ± 17.0  Gen.: 38.4 ± 14.5  after matching: 37.8 ± 13.1 vs  37.8 ± 13.2 | Germany | Brand: 240.5 mg (SD 96.0, range 75–600)  Generic: 209.2 mg (SD 85.1, range 37.5–375)  after matching:  212.1 ± 83.8 (37.5–375) vs  226.1 ± 104.7 (75–450) mg | after matching  Gen: 95.6 ± 52.0 (10–235)  Brand: 93.6 ± 61.7 (13–236) | Gen: 202.2 ± 104.2 (16–476)  Brand: 190.5 ± 98.0 (43–524) | Generic:  320.4 ± 161.0  (26–717)  Brand:  375.5 ± 214.6,  (56–1443)  after matching: Gen: 297.8 ± 148.8 (26–711)  Brand: 284.1 ± 152.1 (56–760) | No differences between generic and brand groups were found. |
| **Unterecker et al. 2014 (Interaction of VA with Dox and Ven)** | CCS with flexible dose design | VEN+VA: 41  VEN: 41  VEN+VA: 45.9±16.2  VEN: 45.7±15.8  *NA* | Germany | VEN+VA: 209.1±81.5 (75–375)  VEN: 206.7±80.0 (75–375) | VEN+VA: 131.1±110.5 (24–488)  VEN: 117.4±76.2 (24–409) | VEN+VA: 271.7±139.6 (52–720)  VEN: 211.1±93.1 (57–516) | VEN+VA: 402.8±210.2 (76–981)  VEN: 328.5±130.4 (115–700) | In patients receiving VEN and valproic acid, the mean serum concentration of ODV was higher than that in patients treated with VEN alone. For C/D of AM, there only was a trend for a difference. |
| **Waade et al. 2014** | CCS with flexible dose design | VEN: 462 samples from 255 patients  *NA* | Norway | range: 18.75–900 | C/D:  Data given in this group order: <40, 40-65, >65 y  2.4 (1.6–3.7) 6.4 (4.7–8.7) 18.8 (9.4–37.6) (PM)  1.7 (1.2–2.4) 2.0 (1.5–2.6) 2.7 (1.8–4.0) (HEM)  1.2 (1.0–1.5) 1.0 (0.8–1.3) 1.4 (0.9–2.1) (EM) | C/D:  Data given in this group order: <40, 40-65, >65 y  1.3 (0.8–2.2) 1.1 (0.7–1.6) 0.6 (0.2–1.3) (PM)  3.2 (2.7–3.9) 3.3 (2.8–3.8) 4.8 (3.8–5.9) (HEM)  3.4 (3.0–3.8) 3.5 (3.1–4.0) 6.1 (4.8–7.8) (EM) | C/D:  Data given in this group order: <40, 40-65, >65 y  3.9 (2.8–5.4) 8.0 (6.2–10.2) 19.7 (11.4–34.2) (PM)  5.3 (4.3–6.5) 5.7 (4.8–6.8) 8.2 (6.5–10.4) (HEM)  5.1 (4.5–5.7) 5.0 (4.4–5.7) 7.9 (6.3–9.9) (EM) | In CYP2D6 PMs, the mean C/D ratio of VEN was about 8-fold and 2.5-fold higher in patients >65 and 40– 65 years, respectively, compared with those <40 years. In comparison, the respective differences in mean C/D ratios of VEN were much less pronounced and not significantly different between CYP2D6 HEMs and EMs . Similar effect of age was observed for VEN+ODV C/D. |
| **van der Weide et al. 2005** | CCS with flexible dose design | VEN: 210 samples of 92 patients  *NA* | Netherlands | NA | NA | NA | NA | There is a significant correlation between the CYP2D6 genotype and the log(VEN/ODV). Gender and age were not significantly different among the 4 genotype groups (P = 0.657 and 0.962 respectively). |
| **Wang et al. 2020** | CCS with flexible dose design | 91 samples from 80  individuals (62,5%)  *multiple diagnosis*  40.1 ± 17.7 | China | 171.4 ± 59.5 | 163.3 ± 136.7  Median:  130.4 (range: 4.9, 888)  C/D:  1.0 ± 0.8  Median (range): 0.8 (0.1, 4.8) | 181.1 ± 93.1  Median:  170.5 (range: 19.4, 527.4)  C/D:  1.1 ± 0.6  Median (range):  1 (0.2, 4) | 341.1 ± 176.3  Median:  317.0 (range: 28.6, 945.0)  C/D:  2.1 ± 1.0  Median (range):  2 (0.5, 5.9) | Remarkable sex differences were found in body weights and BLs of VEN + ODV. Females had an average 13% smaller body weight and an average 43% larger serum concentration of VEN + ODV than males.  It was detected a negative correlation between VEN dosage and the C/D of ODV, and between VEN concentrations and MR, indicating a possible partial saturation of the metabolic capacity. |
| **Warrings et al. 2021** | CCS with flexible dose design | VEN: 380 (58.4%)  *NA*  48.1 ± 16.1 | Germany | 196.29 ± 84.2 (37.5–450) | 130,6 ± 125.1 (5–966)  C/D:  0.70 ± 0.61 (0.04–4.05) | 217.0 ± 135.8 (10–1079)  C/D:  1.18 ± 0.67 (0.09–4.57) | 347.6 ± 212.6 (22–1712)  C/D:  1.88 ± 0.99 (0.15–6.39) | C/D ODV and C/D AM of VEN were negatively associated with BMI. |
| **Ganesh et l. 2021** | CCS  dose design NA | VEN: 193 (65%)  *NA*  Median: 48 | The Netherlands | Median: 131 (IQR: 75 – 150) | NA | NA | NA | A strong correlation between log([VENL]/[DVEN]) ≥ 0.1 and CYP2D6 PM phenotype prediction was found. |
| **RCTs** | | | | | | | | |
| **Boulton et al. 2010**  **Berman et al. 2007 + Marcus et al. 2008 (study 3 + 4 )** | RCT and post hoc analysis with flexible dose design | Total: 498  *Berman et al.*  **Responders:** 262  PLC + VEN: 176  (19 dropouts)  *Marcus et al.*  PLC + VEN: 190  *MDD*  *Berman et al.*  44.2 ± 109  *Marcus et al.*  44.4 ± 10.7 | US | VEN ER (150 - 225 mg/day) | NA | NA | NA | The pooled pharmacokinetic analysis from the two safety and efficacy studies in patients with MDD (Studies 3 and 4) did not demonstrate any meaningful pharmacokinetic effects of VEN on the five ADRs investigated. |

*S11. Table findings from neuroimaging Studies^3^.*

| **References** | **No of subjects (females in %)** | **Mean age ± SD (years)** | **Indication** | **Country** | **Steady state** | **SERT EC80 in ng/mL** | **NET EC50 in ng/mL** |
| --- | --- | --- | --- | --- | --- | --- | --- |
| Arakawa et al. 2019 | 12 (50%) MDD, 9 (70%) HV | MDD: 37.4 ± 11.7  HV: 39.9 ± 14.4 | MDD and HV | Sweden | yes | NA | AM: 671  VEN: 245 |
| Frankle et al. 2017 | 16 (0%) | 27 ± 9 | HV | US | no | ODV: 85 | NA |
| Meyer et al. 2004 | 18 (NA%) | 35 ± 9 | Multiple Dx and HV | Canada | yes | VEN: 13.6 | NA |
| Shang et al. 2007 | 8 (NA%) | 23.6 ± 6.3 | HV | US | yes | NA | NA |

*^3^ Abbreviations used in the data supplement.*

*ADR = Adverse drug reactions*

*ADHD = Attention deficit hyperactivity disorder*

*AM = Active moiety*

*BID = two or more drug administrations per day (altered dose regimen)*

*BL = Blood level*

*C/D = Concentration dose ratio*

*C/E = Concentration/Efficacy*

*CI = Confidence interval*

*CS = Cohort study*

*CSF = Cerebrospinal fluid*

*CSS = Cross sectional study*

*EM = Extensive metabolizer*

*EMC = Early medication change*

*HAMD = Hamilton Depression Rating Scale*

*HV = Healthy volunteers*

*ITT = Intention to treat*

*MADRS = Montgomery–Åsberg Depression Rating Scale*

*MDD = Major depressive disorder*

*MR = Metabolic ratio*

*NA = Not specified*

*NM = Normal metabolizer*

*OCD = Obsessive-compulsive-disorder*

*ODV = O-desmethylvenlafaxine*

*PK = Plasma concentration*

*PM = Poor metabolizer*

*RCT = Randomized controlled trial*

*SCZ = Schizophrenia*

*TAU = Treatment as usual*

*TESI = Treatment emerged suicidal ideation*

*UM = Ultrarapid metabolizer*

*VEN = Venlafaxine*

*WT = Wildtype*

**= manually calculated*

*References:*

McGuinness, L. A., & Higgins, J. P. T. (2020). Risk-of-bias VISualization (robvis): An R package and Shiny web app for visualizing risk-of-bias assessments. *Research Synthesis Methods, n/a*(n/a). doi:10.1002/jrsm.1411
